# Comparative and phylogenomic studies on the mitochondrial genomes of Pentatomomorpha (Insecta: Hemiptera: Heteroptera)

**DOI:** 10.1186/1471-2164-9-610

**Published:** 2008-12-17

**Authors:** Jimeng Hua, Ming Li, Pengzhi Dong, Ying Cui, Qiang Xie, Wenjun Bu

**Affiliations:** 1 Department of Zoology and Developmental Biology, Institute of Entomology, College of Life Sciences, Nankai University, Tianjin 300071, PR China

## Abstract

**Background:**

Nucleotide sequences and the gene arrangements of mitochondrial genomes are effective tools for resolving phylogenetic problems. Hemipteroid insects are known to possess highly reorganized mitochondrial genomes, but in the suborder Heteroptera (Insecta: Hemiptera), there was only one complete mitochondrial genome sequenced without gene rearrangement and the phylogeny of infraorder Pentatomomorpha in Heteroptera was still uncertain.

**Results:**

Fifteen mitochondrial genomes of the suborder Heteroptera were sequenced. Gene rearrangements were found as follows: 1) *tRNA-I *and *tRNA-Q *switched positions in Aradidae, 2) *tRNA-T *and *tRNA-P *switched positions in Largidae and Pyrrhocoridae. Two recombination events were found in Alydidae and Malcidae. The other mt-genomes were organized in the same way as observed in *Drosophila yakuba*. The phylogenetic analyses of infraorder Pentatomomorpha based on the nucleotide sequence raised the hypothesis of (Aradoidea + (Pentatomoidea + (Pyrrhocoroidea + (Lygaeoidea + Coreoidea)))). The rearrangement of *tRNA-T *and *tRNA-P *also linked Largidae and Pyrrhocoridae together. Furthermore, the conserved sequence block in the unusual intergenic spacers between *tRNA-H *and *ND4 *favored the monophyly of Lygaeoidea. Tetranucleotide ATCA was inferred to be the initiation codon of *ND2 *in Cydnidae. No correlation was found between the rates of nucleotide substitution and gene rearrangement. CG content was significantly correlated with the nucleotide substitution rate of each gene. For ND1, there was a positive correlation (*P *< 0.01) between amino acids variations and hydrophobicity, but a negative correlation (*P *< 0.01) for ND6. No conserved sequence was found among the control regions and these regions were not always the most AT-rich region of the mt-genome.

**Conclusion:**

Heteropteran insects are extremely complex groups worthy of further study because of the unusual tetranucleotide initiation codon and their great mt-genomic diversity, including gene rearrangements and recombinations. The mt-genome is a powerful molecular marker for resolving phylogeny at the level of the superfamily and family. Gene rearrangements were not correlated with nucleotide substitution rates. CG content variation caused the different evolutionary patterns among genes. For ND1, in many polar or nonpolar regions the specific identity of the amino acid residues might be more important than maintaining the polarity of these regions, while the opposite is true for ND6. Most sequences of the control regions did not appear to be important for regulatory functions. Finally, we suggest that the term "AT-rich regions" should not be used.

## Background

To date, more than one hundred complete or nearly complete insect mitochondrial genomes (mt-genomes) and thousands of partial sequences are available in GenBank/EMBL/DDBJ, and the data set is expanding at an increasing pace. Most animal mt-genomes contain the same 37 genes, but the orders of the genes are variable among and within the major groups [1]. The mt-genome is becoming an effective tool to resolve phylogenetic problems due to its unique properties, including maternal inheritance, orthologous genes, and lack of the substantial intermolecular recombination [2]. Patterns of mitochondrial gene rearrangements are also indicators of phylogenetic relationships [3-5]

In Insecta, mitochondrial gene rearrangements have been found in 11 orders (including some unpublished data) with the others exhibiting the same gene order observed in *Drosophila yakuba *[6,7]. It has been found that the hemipteroid insects (including four orders, Hemiptera, Thysanoptera, Psocoptera, and Phthiraptera) have experienced increased rates of gene rearrangements in the mt-genomes [8-12]. In the order Phthiraptera, the three sequenced mt-genomes [GenBank: NC002651, GenBank: NC009983, GenBank: NC007884] show different gene arrangements. Within the suborder Sternorrhyncha of order Hemiptera, six of the eight sequenced mt-genomes [GenBank: NC005939, GenBank: NC006159, GenBank: NC006160, GenBank: NC006292, GenBank: NC006279, GenBank: NC006280] experienced gene rearrangements and are different from each other. But within the suborder Heteroptera of order Hemiptera, the gene order of the only sequenced mt-genome, *Triatoma dimidiata *(Latreille, 1811) (Reduviidae) [GenBank: NC002609], is identical to that of *Drosophila yakuba *[GenBank: NC001322].

It has been proposed that such gene rearrangements as synapomorphies would not contribute much to resolving insect interordinal relationships, while using gene order to understand intraordinal relationships would be useful [6]. We undertook to examine whether the gene arrangements are conservative in suborder Heteroptera, especially in the infraorder Pentatomomorpha, to elucidate the phylogenetic relationships within this group and determine whether the gene order could resolve the phylogenetic relationships at the superfamily level.

Pentatomomorpha is one of six infraorders of Heteroptera [13]. It consists chiefly of terrestrial phytophagous bugs. Many of these bugs feed on seeds and plant vascular systems and some feed on fungi. This group of insects is economically important in agriculture and forestry, making robust phylogenetic hypotheses for the main lineages of significant interest. Views on the systematics of Pentatomomorpha before 1993 were summarized by Schaefer (1993) [14]. Currently the classification system of Pentatomomorpha includes five superfamilies, Aradoidea, Pentatomoidea, Coreoidea, Lygaeoidea and Pyrrhocoroidea (the superfamilies except the Aradoidea are grouped as Trichophora) [15]. There are different views of the phylogenetic relationships within Pentatomomorpha based on morphological or molecular evidence. Using morphological evidence, Schaefer (1993) argued that Aradoidea may be either the sister group of Trichophora or Cimicomorpha-cum-Pentatomomorpha [14]. Sweet (1996) proposed that the Aradoidea constituted a separate heteropteran infraorder, Aradomorpha [16]. Henry (1997) suggested the relationship (Aradoidea + (Pentatomoidea + ((Coreoidea + Pyrrhocoroidea) + (Idiostoloidea + Lygaeoidea)))) [17]. Using molecular evidence, Xie et al (2005) performed Bayesian analysis with the 18S rDNA data set on the main lineages of Trichophora, giving the hypothesis of (Pentatomoidea + (Pyrrhocoroidea + (Coreoidea + Lygaeoidea))) and raised the taxon Eutrichophora (including the superfamilies of Trichophora except Pentatomoidea) and Neotrichophora (including Coreoidea and Lygaeoidea), but the relationships within the Neotrichophora were not completely resolved [18]. Li et al. (2005) proposed the hypothesis that (Aradoidea + (Pentatomoidea + ("Pyrrhocoroidea"+ "Coreoidea" + "Lygaeoidea"))) based on the partial sequence of the *CO1 *and 18S rDNA [19]. Li et al. (2005), however, found that the superfamilies Pyrrhocoroidea, Coreoidea, and Lygaeoidea were not monophyletic. However, Li et al. (2005) included several low quality sequences with many ambiguous nucleotides and a lack of subregions, and two CO1-like genes were used instead of the CO1 gene; also, many nodes in the cladogram appeared with bootstrap values lower than 50%. Additionally, Wheeler et al (1993) supported the monophyly of Pentatomomorpha and a sister group relationship between Aradoidea and Trichophora based on evidence combining morphological characters and partial 18S rDNA [20].

To date, the hypothesis (Aradoidea + (Pentatomoidea + the remainder of Trichophora)) has been accepted by most researchers, based on morphological and molecular evidence [17-19,21]. Within Pentatomomorpha, especially the Eutrichophora, the phylogenetic relationships among the superfamilies are still controversial.

For a robust phylogenetic hypothesis of the higher-level relationships in any organism, multiple genes and large sequence lengths are favored [22-24]. However, the DNA sequences that have been used for phylogenetic analyses of Pentatomomorpha are no longer than 1.6 kb. Thus we hoped to resolve the phylogeny of Pentatomomorpha with the mt-genomes, which are the largest set of homologous genes that can be conveniently compared across animal taxa [6]. No bioinformatic studies on the mt-genomes of Pentatomomorpha have been done.

Recombination has been considered to be absent in the animal mt-genome [25], but recombination has been observed in insect mtDNA [26]. There are different views on whether the nucleotide substitution rate correlates with gene rearrangements [27-29].

We sequenced the mt-genomes of 15 heteropterans in this study with five main aims: 1) to explore the organization of their mt-genomes and infer possible evolutionary mechanisms; 2) to find out whether there is recombination in heteropteran mt-genomes; 3) to investigate the phylogenetic relationships among the superfamilies of Pentatomomorpha; 4) to identify the correlation between the nucleotide substitution rate and gene rearrangement; 5) to describe the bioinformatics of heteropteran mt-genomes.

## Results and discussion

A total of 10 complete and 5 nearly complete mt-genomes were sequenced and have been deposited in GenBank (Table 1).

**Table 1:** General informatics of the mt-genomes in this study.

Classification	Species	Mt-genome Completeness	Size (bp)	Mt-genome Type	Total A+T %	AT% of control region	GenBank Accession
**Leptopodomorpha**							
**Saldoidea**							
Saldidae	*Saldula arsenjevi *Vinokurov	complete	15324	A	74.61	77.47	EU427345
**Cimicomorpha**							
**Reduvioidea**							
Reduviidae	*Triatoma dimidiata *(Latreille)	complete	17019	A	69.53	65.77	NC002609*
**Cimicoidea**							
Anthocoridae	*Orius niger *Wolff	*tRNA(I) - 12S*	14494	A	76.53	Incomplete	EU427341
**Pentatomomorpha**							
**Aradoidea**							
Aradidae	*Neuroctenus parus *Hsiao	complete	15354	B	68.86	69.81	EU427340
**Pentatomoidea**							
Pentatomidae	*Nezara viridula *(Linnaeus)	complete	16889	A	76.88	79.45	EF208087
Cydnidae	*Macroscytus subaeneus *(Dallas)	*tRNA(I) - 12S*	14620	A	73.79	Incomplete	EU427338
Plataspidae	*Coptosoma bifaria *Montandon	complete	16179	A	71.33	73.82	EU427334
**Lygaeoidea**							
Berytidae	*Yemmalysus parallelus *Stusak	complete	15747	A	77.18	78.92	EU427346
Colobathristidae	*Phaenacantha marcida *Horvath	*tRNA(I) - 12S*	14540	A	73.46	Incomplete	EU427342
Malcidae	*Malcus inconspicuus *Stys	complete	15575	A	77.80	79.49	EU427339
Geocoridae	*Geocoris pallidipennis *(Costa)	*tRNA(I) - 12S*	14592	A	75.86	Incomplete	EU427336
**Pyrrhocoroidea**							
Largidae	*Physopelta gutta *(Burmeister)	complete	14935	C	74.51	75.45	EU427343
Pyrrhocoridae	*Dysdercus cingulatus *(Fabricius)	complete	16249	C	77.69	79.22	EU427335
**Coreoidea**							
Alydidae	*Riptortus pedestris *Fabricius	complete	17191	A	76.59	76.42	EU427344
Coreidae	*Hydaropsis longirostris *(Hsiao)	complete	16521	A	75.46	73.78	EU427337
Rhopalidae	*Aeschyntelus notatus *Hsiao	*tRNA(I) - 12S*	14532	A	75.71	Incomplete	EU427333

*From Dotson and Beard (2001) [32].

### Genome Organization

Within these genomes, all of the 37 genes commonly found in metazoan mt-genomes were determined: 2 for rRNAs, 22 for tRNAs, and 13 for proteins. Most of these genes are arranged in the same order as the putative ancestral insect mt-genome [30], with a few exceptions discussed later. We also found both intergenic spacers and genes that overlapped by several nucleotides. For discussion purposes, the strand containing the *CO1 *gene was designated as the major-strand (J-strand) because it encoded 23 genes. Consequently the other strand encoding the remaining 14 genes was designated as the minor-strand (N-strand).

**Figure 1:**
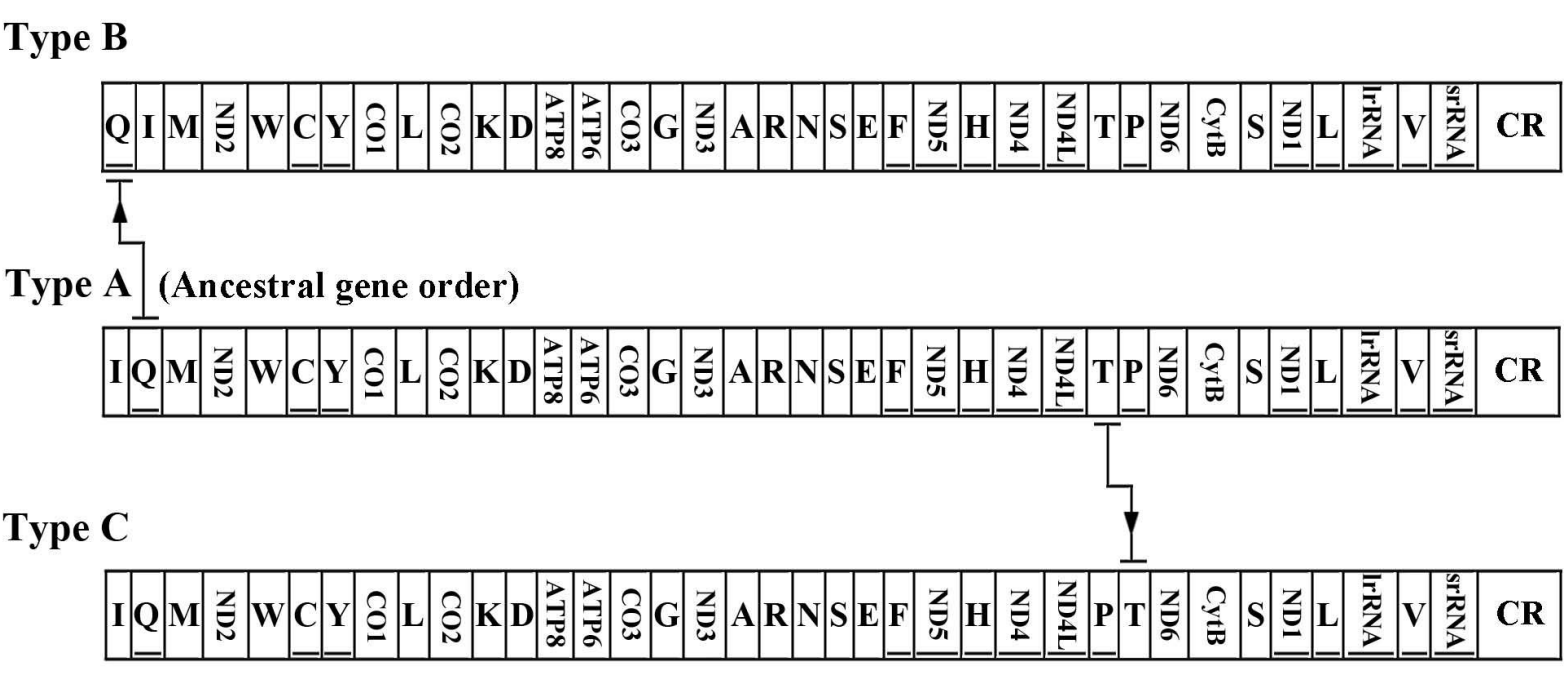
**Types of organization of Heteropteran mt-genomes**. The *tRNAs *were labelled according to the IUPAC-IUB single letter code for the specified amino acid. The underlined genes were encoded on the N-strand.

**Figure 2:**
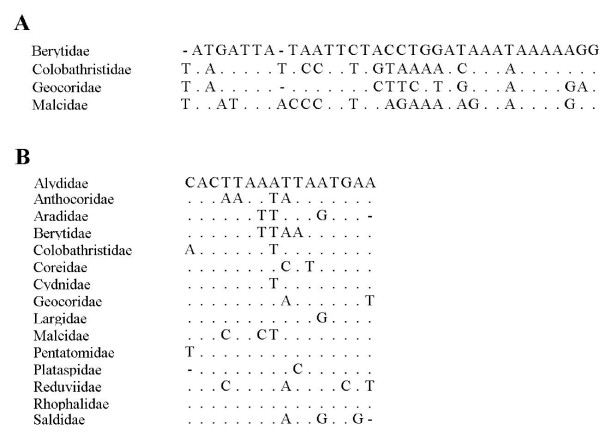
**Alignments of the conserved sequence block (CSB) of the intergenic spacers**. Panel A, the CSB between *tRNA-H *and *ND4 *in Lygaeoidea. Panel B, the CSB between *tRNA-S(TGA) *and *ND1*. The alignments were generated by plotting the identities to a standard as a dot, and a gap as a dash.

Finally, the mt-genomes of heteropterans in this study were classified into three types (A-C) (Table 1 and Figure 1). Because we selected one exemplar species for each representative heteropteran family, the family names are used instead of the species names in the following discussion. Thirteen of the sixteen mt-genomes were classified as type A, with the most common gene arrangement, which contained the identical gene order as the putative ancestral insect mt-genome [30]. Among all the bugs of Lygaeoidea in this study, long intergenic spacers were detected between *tRNA-H *and *ND4 *(38 nt in Berytidae, 40 nt in Malcidae, 72 nt in Colobathristidae, and 124 nt in Geocoridae), which contained a conserved sequence block (CSB) (Figure 2A) that might indicate that the members of Lygaeoidea underwent a common intermediate stage of tandem duplication and random loss (TDRL) [4,31]. In Saldidae and Alydidae, long intergenic spacers were found between *tRNA-I *and *tRNA-Q *(79 nt in Saldidae, 238 nt in Alydidae) but without any CSB.

In type B, Aradidae, the *tRNA-Q *translocated from the inferred ancestral position (*tRNA-I*-and-*tRNA-Q*-and-*tRNA-M*) to upstream of the *tRNA-I *with a 44 nt spacer which could be folded as a stem-loop structure between them.

In most of the sequenced mt-genomes of heteropterans, *tRNA-T *and *tRNA-P *form a concatenated unit and locate upstream to *ND6 *gene. But in Type C, Pyrrhocoroidea (including Largidae and Pyrrhocoridae), *tRNA-T *and *tRNA-P *switched their positions with two extra intergenic spacers in different locations. In Largidae, the spacers are 19 nt between *ND4L *and *tRNA-P*, and 240 nt between *tRNA-T *and *ND6*. In Pyrrhocoridae, the spacers are 33 nt between *ND4L *and *tRNA-P*, and 60 nt between *tRNA-P *and *tRNA-T*.

Furthermore, the intergenic spacer between *tRNA-S (TGA) *and *ND1 *occurs in all the mt-genomes except Pyrrhocoridae. All these spacers are no longer than 30 nt except Reduviidae which is longer than 300 nt. Some of these spacers may be folded as stem-loop structures, and in Reduviidae this spacer contains an unidentified open reading frame of 312 nt [32]. Among these spacers, there is a CSB (Figure 2B), which might indicate that the extant heteropterans underwent a common intermediate stage of the TDRL process, i.e., this spacer might be a synapomorphy of heteropterans.

### Possible evolutionary mechanisms of gene rearrangement

**Figure 3:**
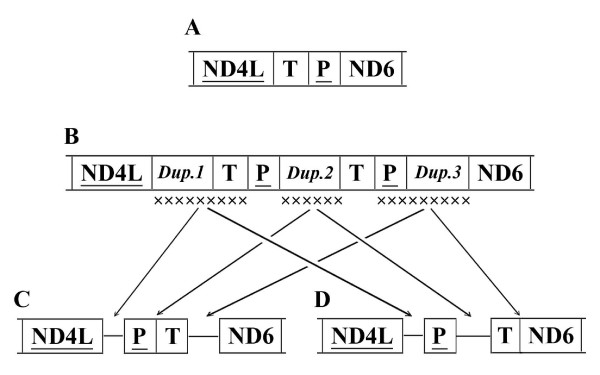
**Possible evolutionary mechanism of the gene rearrangement in Largidae and Pyrrhocoridae**. A, the putative ancestral arrangement; some regions of mtDNA including the *tRNA-T *and the *tRNA-P *were duplicated to form the intermediate (B) where some regions were lost to form C, Largidae D, Pyrrhocoridae. *Dup.1-3 *are the duplicated regions. The X in B indicates the randomly lost regions.

The translocated *tRNAs *in Aradidae, Largidae, and Pyrrhocoridae have not changed their transcription direction. Although it is unknown what really happened to these mt-genomes causing the gene rearrangements and intergenic insertions, we tried to elucidate the possible evolutionary mechanisms using the most commonly supposed model for the gene rearrangements, including gene tandem duplication and random loss [4,31]. The possible mechanism of position switch of *tRNA-T *and *tRNA-P *in Largidae and Pyrrhocoridae is shown in Figure 3. The TDRL shown from Figure 3A (ancestral state) to Figure 3C (Largidae) and Figure 3D (Pyrrhocoridae) might include several different pathways. For example, the duplication of the *Dup.2 *is not necessary to produce Figure 3C and the *Dup.3 *is not necessary to Figure 3D, i.e., Largidae and Pyrrhocoridae might have been formed separately. However, because convergent rearrangements are rare in mt-genomes [33], and Largidae and Pyrrhocoridae are grouped as the monophyletic superfamily Pyrrhocoroidea (discussed later), these two rearrangements of *tRNA-T *and *tRNA-P *are speculated to be the products of the same intermediate and they are synapomorphies of the members of Pyrrhocoroidea. The translocation of the *tRNA-Q *in Aradidae could be elucidated with the same model.

### Recombination of mitochondrial DNA

**Figure 4:**
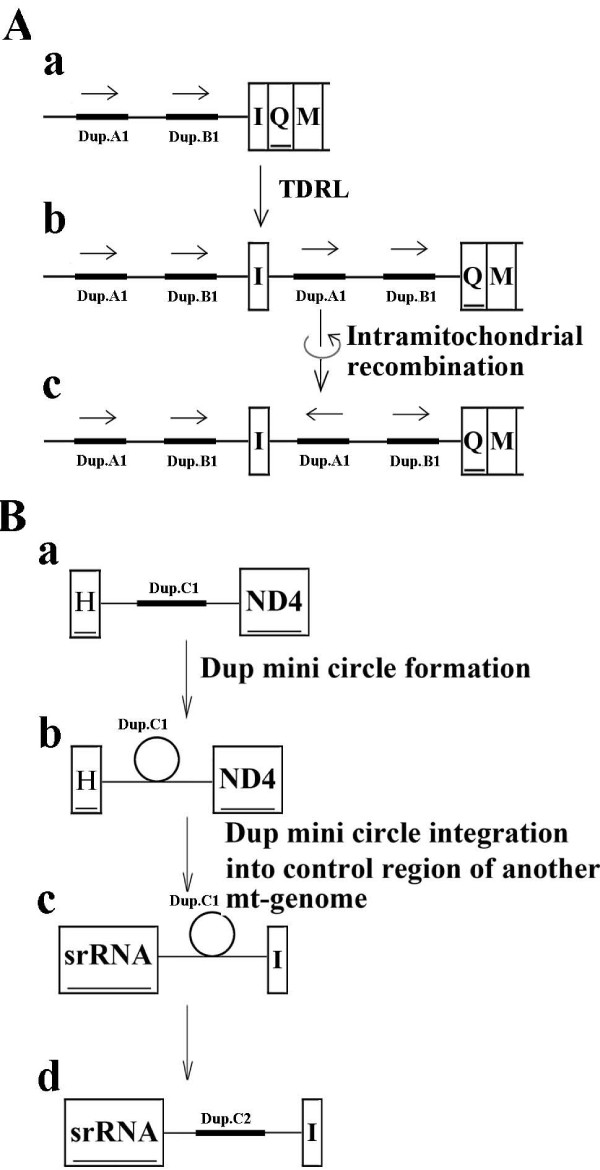
**Possible evolutionary mechanism of recombination in Alydidae and Malcidae**. A, recombination in Alydidae. Panel A.a, the ancestral state. Dup.A1 and Dup.B1 were two original copies of the repetitions. Panel A.b, Dup.A2 and Dup.B2 between *tRNA-I *and *tRNA-Q *were repetitions of the Dup.A1 and the Dup.B1 formed by the tandem duplication and random loss. Panel A.c, Dup.A2 changed its direction by intramitochondrial recombination [35], forming the extant state. B, the recombination in Malcidae. Panel B.a, Dup.C1 was the original copy of thr repetition in the intergenic spacer between *tRNA-H *and *ND4*. Panel B.b, formation of the mini circle of Dup.C1 by intramitochondrial recombination [35]. Panel B.c, the mini circle of the Dup.C1 integrated into the control region of another mt-genome. Panel B.d, Dup.C2, the repetition of the Dup.C1, formed in the control region.

In Alydidae, two subregions of the intergenic spacer between the *tRNA-I *and the *tRNA-Q *have a repeated counterpart (29 nt, with one site mutation, Blast E-value: 3e-9) and an exactly inverted repeated counterpart (26 nt, Blast E-value: 8e-10) in the control region (Figure 4A) (see Additional file 1). Since the TDRL model would only produce tandem duplications [34], these two repetitions are supposed to be formed by the TDRL following an intramitochondrial recombination [35] as shown in Figure 4A.

In Malcidae, one subregion of the intergenic spacer between the *tRNA-H *and the *ND4 *has an exactly repeated counterpart in the control region (34 nt, Blast E-value: 2e-15) (see Additional file 1), and this subregion is also part of the CSB in Lygaeoidea. Considering that this intergenic spacer is shared among the members of Lygaeoidea, in Malcidae the copy of the repetition located between *tRNA-H *and *ND4 *is supposed to be the original one. This phenomenon could also be elucidated by the TDRL model wherein a large fragment from the intergenic spacer to the control region duplicated and then most of them were lost except the repetition. However, across the whole mt-genome, no vestige of such a large duplication was found. The possible alternative mechanism is recombination. It has been reported that the secondary structures formed by the tRNA genes or the self complementary sequences may play a major role as the hotspots in recombination [36,37], and the control region is supposed to be a possible recombination hotspot [27]. Interestingly, this intergenic spacer in Malcidae is capable of forming a stem-loop structure (see Additional file 1) adjacent to *tRNA-H*, and its repetition in the stem-loop-rich control region is adjacent to *tRNA-I*. So, we speculated that this duplication might be the product of illegitimate recombination via a mini-circle (Figure 4B). However, in Berytidae, no copy of this spacer was found across the mt-genomes. Further study is needed to investigate whether this repetition is the autapomorphy of Malcidae, since the control regions of Geocoridae and Colobathristidae could not be sequenced because of poly A and poly T.

### Use of the tRNAs in phylogenetic analysis

The transfer RNA gene recruitment or the duplication and the remolding make it hard to identify the homologs for these short genes and could also affect the use of mitochondrial gene orders for the phylogenetic reconstructions [38,39]. It has also been proposed that the *tRNAs *are much less homoplastic than the PCGs and should be included in the phylogenetic analyses [6]. Thus, it is necessary to understand whether the tRNA genes are homologs before the phylogenetic analyses.

For the 22 tRNAs, each type holds the same anticodon respectively across the mt-genomes in this study. We aligned each type of *tRNA *and adjusted them according to their secondary structures (the alignments are the same as those used in phylogenetic analyses), and then the pairwise similarities within each type of *tRNA *were calculated by pairwise alignment using BioEdit (see Additional file 2). High similarity values could found within each type of the *tRNAs*, indicating that no *tRNA *recruitment or remolding [38,39] happened and the rearrangements of the *tRNAs *in Aradidae, Largidae, and Pyrrhocoridae should be the transpositions of the tRNA coding sequences. Finally, we determined that each group of *tRNAs *used in the phylogenetic analyses are homologous.

### Phylogeny of Pentatomomorpha

**Figure 5:**
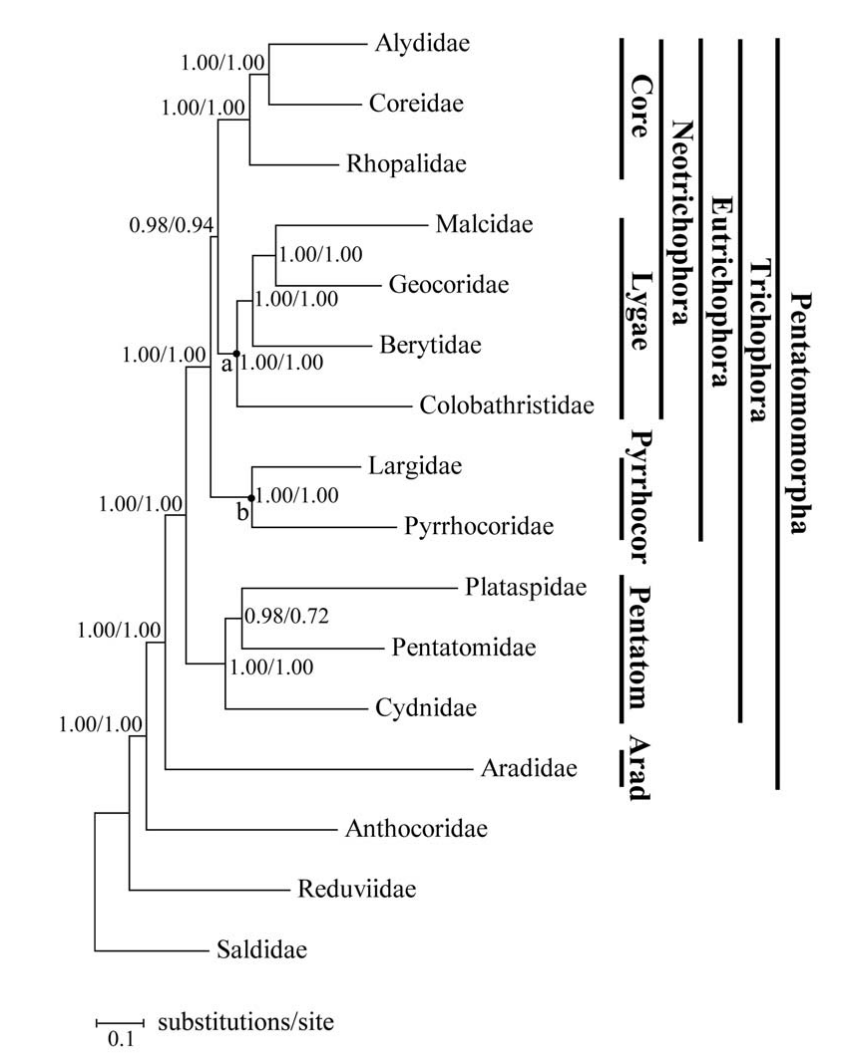
**Phylogram of Pentatomomorpha from Bayesian inference of the N123RT data set**. The tree from the ML analyses and Bayesian inference with the N123RT data set and N12 data set showed the same topology. The Bayesian posterior probabilities are indicated at each node. The node "a" is also supported by the CSB between *tRNA-H *and *ND4*. Node "b" was also supported by the rearrangements of *tRNA-T *and *tRNA-P*. On the right, the name of each superfamily is abbreviated by removing the postfix -oidea.

The N123RT data set contained 15322 nucleotides sites and the N12 data set contained 7516 nucleotides sites for each of the 16 taxa. Finally, all four phylogenetic analyses conducted with Bayesian inference and ML received fully bifurcating trees with the same topology rooted with Saldidae (Figure 5). All the superfamilies established previously in Pentatomomorpha are well recognized. Significantly, our results support the hypothesis that Pentatomoidea, Pyrrhocoroidea, Lygaeoidea, and Coreoidea are monophyletic groups. Though only one species was chosen in Aradoidea, up to now no one has challenged the monophyly of Aradoidea [17].

Our results also confirm that Aradoidea and the Trichophora are sister groups as indicated in previous studies [17-19,21]. Hence, we argue that there is no necessity to raise Aradoidea from a superfamily to an infraorder, because the seven-infraorder classification of the Heteroptera has been accepted by most researchers [15,20]. The sister groups' relationship of Pentatomoidea and the remainder of the Trichophora raised in this study also conforms to the morphological and nuclear rDNA data [17,18]. In Eutrichophora, our study raises a phylogenetic hypothesis of (Pyrrhocoroidea + (Lygaeoidea + Coreoidea)). This result is different from Henry (1997) [17] but similar to Xie et al (2005) [18]. Since this study did not include all the families of each superfamily, family relationships within each superfamily, remain incomplete. The Rhophalidae presents a sister position to the (Coreidae+Alydidae) and the monophyly of the (Coreidae+Alydidae) is also accepted by Henry (1997) [17] and Xie et al (2005) [18]. In Lygaeoidea, our conclusion is (Colobathristidae + (Berytidae + (Geocoridae + Malcidae))), which is different from Henry (1997) [17], whose hypothesis could be summarized as (Geocoridae + (Malcidae + (Colobathristidae + Berytidae))). The monophyletic malcid line [17,40] is broken down in our study because of the interposition of Geocoridae.

Surprisingly, the infraorder Cimicomorpha (including Reduviidae, Anthocoridae, and other families not included in this study), whose monophyly has been accepted [20,41], was not monophyletic in this study. The Bayesian posterior probability value for the node (Anthocoridae + Pentatomomorpha) is 1.00. The mt-genome data in this study may be too limited to resolve the phylogeny of Cimicomorpha, and more mt-genomes of the additional taxa of Cimicomorpha need to be sequenced because increased taxon sampling could greatly reduce the phylogenetic error [42], or possibly the monophyly of Cimicomorpha needs to be revised.

Additionally, in Lygaeoidea the CSB in the intergenic spacers between *tRNA-H *and *ND4 *indicates that the members of Lygaeoidea have undergone a common intermediate TDRL and this spacer is the synapomorphy of Lygaeoidea favoring its monophyly. Furthermore, the position switch of *tRNA-T *and *tRNA-P *also links Largidae and Pyrrhocoridae together.

### Correlation between rates of nucleotide substitution and gene rearrangement

It has been found that the substitution rate is not relevant to the frequent gene rearrangements in the pond-breeding mantellids [27]. However, a positive correlation has been demonstrated in the arthropod taxa [28,29]. To find this correlation in Pentatomomorpha, the relative nucleotide substitution rates were tested among three groups: 1) Aradidae, whose *tRNA-I *and *tRNA-Q *switched positions; 2) Largidae and Pyrrhocoridae, whose *tRNA-T *and *tRNA-P *switched positions; and 3) the remainder of Pentatomomorpha (Table 2). For *tRNA-Q*, Aradidae has a significantly different substitution rate from other two groups. For *tRNA-P*, Largidae and Pyrrhocoridae have significantly different substitution rates. For *tRNA-T*, the three groups present significantly different substitution rates from each other. However, for *tRNA-I*, Largidae and Pyrrhocoridae, whose *tRNA-Is *do not translocate, present significantly different substitution rates, but not the Aradidae. Interestingly, among all the *tRNA-P*, Pyrrhocoridae is the most TA-skewed and GC-skewed, and among all the *tRNA-T*, Largidae is the most AT-skewed and CG-skewed (Table 3). Further studies are needed to say why these two tRNA genes present different substitution patterns from their homologues and whether the increased mutations are accumulated before or after the rearrangements

**Table 2:** Relative nucleotide substitution rate test among the lineages of Pentatomomorpha.

Comparison	La	Lb	Z
**All genes**			
A/B	0.252	0.1763	14.5532 *
A/C	0.2504	0.1779	15.4327 *
B/C	0.1579	0.161	1.16867
**Total *tRNAs***			
A/B	0.1447	0.1315	1.04073
A/C	0.1366	0.1146	2.1035 *
B/C	0.1119	0.103	1.21956
***tRNA-I***			
A/B	0.1753	0.07057	1.87305
A/C	0.1412	0.07248	1.30467
B/C	0.06795	0.1039	2.28988 *
***tRNA-Q***			
A/B	0.3004	0.07951	2.5092 *
A/C	0.3157	0.1326	2.17766 *
B/C	0.08341	0.1212	1.34169
***tRNA-P***			
A/B	0.1192	0.2319	1.37894
A/C	0.1965	0.1125	1.32347
B/C	0.2633	0.06678	2.72441 *
***tRNA-T***			
A/B	0.02991	0.2118	3.69325 *
A/C	0.02602	0.07338	2.27682 *
B/C	0.1953	0.0608	3.3337 *

Note. A, Aradoidea; B, Largidae and Pyrrhocoridae; C, the remainder of Pentatomomorpha; Anthocoridae, Reduviidae, and Saldidae were used as the outgroup. La and Lb, the averages of observed numbers of substitutions per site (branch lengths) from the outgroup of two clades. Z, the parameter calculated by Phyltest [71]. The analyses were carried out under the Kimura-2-parameter distances. The asterisks mark the significantly different substitution rates (*P *< 0.05).

**Table 3:** Nucleotide composition of the *tRNA-T *and the *tRNA-P*.

	*tRNA-T*			*tRNA-P*		
		
	AT%	AT-skew*	CG-skew**	AT%	AT-skew*	CG-skew**
Alydidae	0.841	0.094	-0.200	0.806	-0.040	-0.500
Anthocoridae	0.833	0.055	-0.091	0.769	0.000	-0.467
Aradidae	0.803	0.020	-0.167	0.677	-0.045	-0.333
Berytidae	0.785	0.059	0.000	0.742	-0.043	-0.375
Colobathristidae	0.855	0.057	-0.111	0.794	-0.120	-0.385
Coreidae	0.813	0.077	0.000	0.785	-0.059	-0.429
Cydnidae	0.846	0.055	-0.200	0.797	-0.020	-0.538
Geocoridae	0.794	0.040	-0.231	0.762	-0.083	-0.467
**Largidae**	0.818	**0.148**	**0.167**	0.781	-0.120	-0.571
Malcidae	0.803	0.143	0.000	0.800	-0.125	-0.500
Pentatomidae	0.769	0.080	-0.067	0.778	-0.020	-0.429
Plataspidae	0.824	0.036	0.000	0.719	-0.087	-0.333
**Pyrrhocoridae**	0.797	0.137	-0.077	0.703	**-0.200**	**-0.579**
Reduviidae	0.818	0.111	-0.167	0.735	-0.080	-0.444
Rhopalidae	0.810	0.059	-0.167	0.815	-0.094	-0.500
Saldidae	0.762	0.042	-0.067	0.797	-0.098	-0.538

* and **, the nucleotide skew statistics [55], AT-skew = (A-T)/(A+T), CG-skew = (C-G)/(C+G).

In general, the groups whose genes rearranged do not always exhibit significantly different substitution rates. So, there is no correlation between the rates of nucleotide substitution and gene rearrangement.

### Different evolutionary patterns among genes

The nucleotide substitution rates averaged over all sequence pairs of each gene vary among genes (see Additional file 3A). The overall mean value of this ratio of each pair of PCGs is 0.639, while for the *rRNAs *it is 0.384. Furthermore, the concatenated 22 *tRNAs *present a lower ratio (0.241) than PCGs and *rRNAs*, as reported before [43]. This is a reasonable result because the purifying selection constrains nucleotide divergence in the rRNAs and the tRNAs due to the secondary structures of these molecules and the specificity of their amino acid binding sites during the translation [44]. *ND6 *presents the highest nucleotide substitution rate, while *CO1 *appears to be the lowest. Surprisingly, *CO2*, which is considered as one of the slowest evolving genes [45], presents a higher nucleotide substitution ratio than most of the PCGs. As shown in Additional file 3A, the number of synonymous substitutions per synonymous site of *CO2 *is the highest, but its number of nonsynonymous substitutions per nonsynonymous site is much lower, while this value for *ATP8 *is the highest. As far as the PCGs are concerned, at the amino acid level the cytochrome oxidase subunits (CO1, CO2, and CO3) and cytochrome b are the slowest evolving proteins and ATP8 is the fastest. The nucleotide substitution rates of the first and second codon positions of PCGs are always lower but more variable within each gene than the third codon positions (see Additional file 3B), which is most likely because of the different selective constraints on the different genes [46]. Obviously, for PCGs the ratios of the nonsynonymous nucleotide changes (Ka) versus the synonymous nucleotide changes (Ks) are all below 1, indicating that they are evolving according to the purifying selection [47].

Negative correlations have been found between the nucleotide substitution rate and the CG content of each gene (*P *< 0.01), and between the Ka/Ks and the CG content of each PCG (*P *< 0.01), which indicate that the variation of CG content causes the different evolutionary patterns among genes. For ND1, there is positive correlation (*P *< 0.01) between the variations and the hydrophobicity of the amino acids, but negative correlation (*P *< 0.01) for ND6, indicating that, in many polar or nonpolar regions, the specific identify of amino acid residues may be more important than maintaining the polarity of these regions of ND1, while the opposite is true for ND6, as reported in other organisms [48].

### Codon usage and nucleotide composition

**Figure 6:**
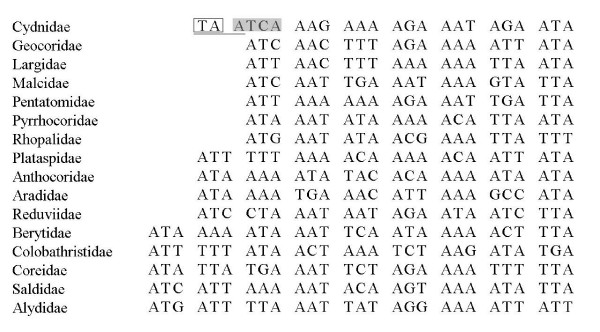
**Alignments of the initiation subregion of *ND2***. The sequences were divided according to the triplet. In Cydnidae, the underlined TAA was the stop codon in the same ORF located upstream adjacent to the possible initiation position; the boxed TA is the 3'-end of the *tRNA-M*; the shadowed tetranucleotide ATCA is the supposed initiation codon.

Most of the PCGs in the mt-genomes of heteropterans are initiated with the common triplet initiation codons as shown in the invertebrate mitochondrial genetic code table. However, in mt-genomes of insects, atypical initiation codons are not rare, e.g., the tetranucleotide TTAG serves as the initiation codon for *Bombyx mori *(Lepidoptera: Bombycidae) [49], and ATAA, GTAA, TTAA for *Drosophila *(Diptera: Drosophilidae) [7,50-52]. In Cydnidae, *ND2 *was supposed to be initiated with an atypical initiation codon, ATCA, based on the alignments with the other heteropterans (Figure 6).

Some PCGs do not possess complete stop codons but appear to be terminated with a single T adjacent to a downstream tRNA gene on the same strand (data not shown, see the GenBank files under the Accession Numbers in Table 1). This is not peculiar the in mt-genome and a TAA stop codon is probably generated by posttranscriptional polyadenylation [53]. RNase P has been found to take on some aspects of the structural conformation of the tRNAs for the recognition signals of the cleavage sites in the tRNA precursors, rather than the nucleotide sequences [53]. Thus, we supposed that the site right before tRNA should be the cleavage site. However, the complete common stop codons always overlap several nucleotides within the downstream *tRNA*, which perhaps act as a "backup" to prevent the translational read through if the transcripts are not properly cleaved [54].

In Heteroptera, the nucleotide compositions are biased toward adenines and thymines (68.86% to 77.8% of A+T) (Table 1). The nucleotide skew statistics [55] of J-strands indicate that the heteropterans are AT-skewed and CG-skewed (Table 4). Although several mt-genomes are not completely sequenced, these skew trends are obvious. The skew statistics of the total PCGs demonstrate that the N-strand PCGs are GC-skewed and much more TA-skewed while the J-strand PCGs are CG-skewed and consist of nearly equal A and T. It is possible that the overall genome A-bias is driven by mutational pressure on the N-strand [56] and the GC-skew may be correlated with the asymmetric replication process of the mtDNA [45].

**Table 4:** Nucleotide composition of the mt-genomes in this study

Taxa	Length of total genes (bp)	Length of control region (bp)	Total J-strand		PCGs of J-strand		PCGs of N-strand	
					
			AT-Skew	CG-Skew	AT-Skew	CG-Skew	AT-Skew	CG-Skew
Saldidae	14593	697	0.158	0.141	0.056	0.108	-0.353	-0.164
Reduviidae	14572	2165	0.168	0.265	0.002	0.220	-0.427	-0.349
Anthocoridae	14527	Incomplete	0.159	0.101	0.063	0.071	-0.335	-0.125
Aradidae	14687	649	0.199	0.220	0.104	0.201	-0.398	-0.275
Pentatomidae	14641	2190	0.125	0.149	0.024	0.073	-0.305	-0.200
Cydnidae	14642	Incomplete	0.126	0.158	0.017	0.091	-0.298	-0.239
Plataspidae	14597	1585	0.147	0.130	0.064	0.097	-0.349	-0.111
Berytidae	14546	1181	0.091	0.098	-0.014	0.012	-0.295	-0.151
Colobathristidae	14475	Incomplete	0.206	0.190	0.095	0.174	-0.398	-0.211
Malcidae	14399	1180	0.138	0.194	0.013	0.145	-0.319	-0.250
Geocoridae	14471	Incomplete	0.142	0.147	0.025	0.094	-0.328	-0.203
Largidae	14459	224	0.206	0.207	0.107	0.168	-0.408	-0.300
Pyrrhocoridae	14555	1617	0.135	0.220	0.012	0.139	-0.334	-0.310
Alydidae	14553	2400	0.093	0.193	-0.040	0.104	-0.253	-0.238
Coreidae	14534	1991	0.102	0.253	-0.034	0.152	-0.295	-0.322
Rhopalidae	14553	Incomplete	0.132	0.168	0.005	0.092	-0.325	-0.282

Because there are only 22 tRNA genes in one mt-genome, one tRNA must recognize more than one type of codon. However, the most frequent codons do not always correspond with their cognates in the mitochondrial tRNAs (see Additional file 4). It appears that the recognition of the cognates between the anticodons and the codons is not related to translation efficiency. Furthermore, the most infrequently used fourfold degenerate codons are NNG, which is because in the oxidative conditions of the mitochondria, the low content of the less stable G acts on the base composition of the mRNAs to preserve their life span, rather than the DNA [57]. Overall, the most frequently used codons are NNA, which could be explained by the hypothesis put forward by Xia (1996)[71] that ATP is much more abundant than the other three ribonucleotides in mitochondria, and the most frequently used ribonucleotide at the third codon positions in the mRNA should be the same as the most abundant ribonucleotide in the cellular matrix where the mRNA is transcribed [58].

### tRNAs and rRNAs

Based on the DNA sequences, most of the tRNAs could be folded as cloverleaf secondary structures. A few of them possessed non-Watson-Crick matches, aberrant loops, or even extremely short arms. Most of the tRNA-S (GCT) lost their DHU arms, which is not rare in insect mt-genomes. In some cases, the alternative folding could yield a structure with an extremely short DHU stem and a small loop. It has been supposed that these characteristics in mt-genome are partly because the mtDNA is not subject to the process of recombination, which may facilitate the elimination of deleterious mutations [59]. However, recombination in insect mtDNA has been observed [26]. It is not known whether the aberrant tRNAs lose their function in every case, but there are reports of the recruitment of nuclear tRNAs into the mitochondria [60,61], and a type of RNA editing could recover the well-paired acceptor stem [62].

Since it is impossible to precisely determine the ends of rRNA genes by DNA sequence alone, they were assumed to extend to the boundaries of flanking genes [3,54]. So the *lrRNA *was assumed to fill up the blanks between *tRNA-V *and *tRNA-L (TAG)*. For the boundary between the *srRNA *gene and the non-coding putative control region, alignments with homologous sequences in other insect mt-genomes were applied to define the 5'-end of the gene [11,63,64].

### Noncoding sequences

Sizes of the complete mt-genomes of Heteroptera range from 14935 bp (Largidae) to 17191 bp (Alydidae) (Table 1). Total length of *tRNAs*, *rRNAs*, and PCGs of each mt-genome does not vary too much in this study, ranging from 14399 bp (Malcidae) to 14687 bp (Aradidae) (Table 4). So the variety of mt-genome size is mainly derived from the non-coding regions. Among these mt-genomes, some large intergenic spacers (the control regions located between *srRNA *and *tRNA-I *are not included) could be folded as stem-loop structures which may be potential control elements, and several short ORFs could be found with no significant similarity to any known insect proteins. Since mt-genomes without these "control elements" or "proteins" could still preserve their life, these spacers were supposed to be vestiges of their common or their own ancestral intermediates generated by gene duplication-random loss process of rearrangement, so were the other short spacers.

As summarized by Cook (2005), the putative control regions of the arthropods often have any or all of these four motifs: a long sequence of thymines, tandemly repeated sequences, a subregion of even higher A+T content, and stem-loop structures [65]. Among the 11 complete mt-genomes of heteropterans, lengths of the control regions range from 224 bp (Largidae) to 2400 bp (Alydidae) (Table 4). Besides the stretches of thymines in all of them, only some short tandemly repeated sequences could be found in several of the mt-genomes, such as the (TA)_6 _in Saldidae, the (TTGA)_3 _in Aradidae, the (AAATC)_2 _in Coreidae, and the (TTAA)_3 _twice separately in Malcidae. Interestingly, Alydidae, whose control region is the longest one in this study, had only several short tandemly repeated sequences no longer than 11 bp (see Additional file 5). In Reduviidae [32], Berytidae, Largidae, Pentatomidae, Plataspidae, and Pyrrhocoridae, several repetitions larger than 100 bp have been found. All of the large repetitions and some large non-repetitions could be folded into stem-loop structures. However, no CSB has been found among these control regions. Because the control regions show extreme variability across the taxa and even among closely related species, primary sequence of much of the control region does not appear to be particularly important for regulatory function [48]. Among these 11 complete heteropteran mt-genomes, the control regions are not always the most AT-rich regions (Table 1). Thus the common alternative term for the control region, the AT-rich region, should probably not be used even though each control region had one or several more AT-rich subregions. Interestingly, in most cases the large tandemly repeated sequences are more AT-rich but not always the most, especially in Plataspidae, where large tandem repetitions consisting of (117 bp)_4 _are the most AT-rich subregions (calculated from J-strand) (see Additional file 6).

## Conclusion

In this study, the data sets of mt-genomes in Heteroptera were enlarged. These true bugs experienced a high rate of the genome reorganization; translocations of *tRNAs *were found in Aradidae, Pyrrhocoridae, and Largidae, recombination events were observed in Alydidae and Malcidae, and a tetranucleotide ATCA was inferred to be the initiation codon of *ND2 *in Cydnidae. So, considering the mt-genomic sequences developed in previous studies, the hemipteroid insects are extremely complex groups exhibiting great mt-genomic diversity and these insects are well worth further study.

In infraorder Pentatomomorpha, phylogenetic analyses based on nucleotide sequences favor the monophyly of each superfamily (the monophyly of Aradoidea has been generally accepted though it has not been analyzed in this study) and raise a robust hypothesis of (Aradoidea + (Pentatomoidea + (Pyrrhocoroidea + (Lygaeoidea + Coreoidea)))). Rearrangement of *tRNA-T *and *tRNA-P *also favor the monophyly of Pyrrhocoroidea. Furthermore, the CSB in the intergenic spacers between *tRNA-H *and *ND4 *favor the monophyly of Lygaeoidea.

Nucleotide sequence of mt-genome is a powerful molecular tool for resolving insect phylogenetic relationships at the level of the superfamily and family. However, use of gene arrangement for phylogenetic analysis in such hierarchies is limited because of its rarity. In future studies, larger taxa sampling is needed to establish the feasibility of using gene arrangement for phylogenetic analysis.

Gene rearrangements are not correlated with nucleotide substitution rates. Different evolutionary patterns among the genes are derived from the variation of the CG content. CO1 is the slowest evolving protein and ATP8 is the fastest one. For ND1, in many polar or nonpolar regions, the specific identify of amino acid residues may be more important than maintaining the polarity of these regions, while the opposite is true for ND6. No CSB has been found among the control regions and many parts of the control regions do not appear to be particularly important for regulatory functions. The use of the term "AT-rich region" to describe the control region should be reconsidered, because this region is not always the most AT-rich part of the mt-genome.

## Materials and methods

### Taxon Sampling

Thirteen exemplar species were sampled in the infraorder Pentatomomorpha, which represented 13 families of Pentatomomorpha and one species of Leptopodomorpha, and two species (one from GenBank) of Cimicomorpha (Table 1) were chosen as the outgroups according to Wheeler et al (1993) [20] and Schuh and Slater (1995) [15].

### Material preparation

Adult specimens used in this study were collected in Yunnan Province, Guizhou Province and Tianjin City, China, in recent years. All specimens were preserved in 95% ethanol in the field. After transport to the laboratory, they were stored at -20°C until DNA extraction.

### Mitochondrial DNA preparation, amplification and sequencing

Total genomic DNA was extracted from thorax muscle tissue using a CTAB-based method [66]. The mt-genomes were amplified with two to four overlapped fragments. The primers of co1F, cytbF, and cytbR were modified from Simon et al. (1994) [67]. The other primers were then designed from the sequenced fragments of the *CO1 *and the *Cytb *amplified with co1F & co1R and cytbF & cytbR or from the comparison results of the *CO1*, the *CO2*, and the *Cytb *of other insects. Each pair of primers special to each species to amplify two long fragments were named with the first three letters of the family name (see Additional file 7). PCR reactions were performed with a TaKaRa LA PCR Kit Ver.2.1 according to the manufacturer's recommendations. PCR thermal cycling included 1 minute initial denaturation at 94°C, 32 cycles of 20 seconds at 94°C, 1 minute at 50°C~60°C, and 1~10 minutes at 68°C, and a final elongation for 15 minutes at 72°C. The PCR products were electrophoresed in 0.7% agarose gel and purified, then sequenced on both strands with the primer walking method by Beijing Sunbiotech Co. Ltd.

### Sequence assembly and analysis

Sequence assembly was done using BioEdit version 7.0 [68]. Protein coding genes (PCGs) were identified by ORF Finder implemented by the NCBI website  with the invertebrate mitochondrial genetic codes and comparison with the published insect mitochondrial sequences with CLUSTAL X version 1.83 [69]. Ribosomal RNA genes were assumed to extend to the boundaries of flanking genes and were also compared with the published insect mitochondrial sequences with CLUSTAL X. Transfer RNA analysis was conducted using tRNAscan-SE version 1.21 [70] with the invertebrate mitochondrial codon predictors, and the tRNAs not detected by tRNAscan-SE were identified by BLAST search and comparison with the other determined heteropterans in this study. Analyses of sequences were performed with Phyltest [71], MEGA version 4.0 [72], DnaSP version 4.10.9 [73] and DAMBE version 4.2.13 [74,75].

### Phylogenetic analysis

Complete sequences of each gene were used in the phylogenetic analyses except the stop codons of the protein coding genes. All PCGs were aligned based on amino acid sequences aligned with MEGA, *rRNAs *and *tRNAs *were aligned with CLUSTAL X under the default settings. The ambiguously aligned regions of PCGs and rRNA genes were carefully adjusted "by eye". The alignments of tRNA genes were corrected according to the secondary structures, especially the stem regions. Then the aligned sequences were concatenated to be the N123RT data set including 2 *rRNAs*, 22 *tRNAs *and 13 PCGs. This data set was used to reconstruct the phylogenetic trees by ML and Bayesian analyses respectively. In both of the algorithms, the general reversible model (GTR + I + G) and the parameters optimized by Modeltest version 3.7 [76] were used. ML analyses were conducted with PAUP* version 4.0b10 [77]. Bayesian analyses were conducted with MrBayes version 3.1.1 [78], with two simultaneous runs for 1,000,000 generations with the first 25% discarded as burn-in. Another data set including only the 1st and 2nd codon positions of PCGs (the N12 data set) was also used to reconstruct the phylogeny by the same methods.

## Abbreviations

*CO1*, *CO2*, and *CO3*: cytochrome c oxidase subunit I, II, and III genes; *CytB*: cytochrome b gene; *ATP6 *and *ATP8*: ATP synthase F0 subunit 6 and 8 genes; *ND1*, *ND2*, *ND3*, *ND4*, *ND4L*, *ND5*, *ND6*: NADH dehydrogenase subunit 1–6 and 4L genes; transfer RNA genes were labeled according to the IUPAC-IUB single letter code for the specified amino acid; in cases where there was more than one tRNA for a particular amino acid, they were distinguished by their anticodons.

## Authors' contributions

JH designed the experiments, carried out the data analyses and drafted the manuscript. ML and PD participated in the experiments. YC helped to prepare the additional files. QX helped to draft the manuscript. WB directed this study and revised the manuscript. All authors read and approved the final manuscript.

## Supplementary Material

Additional file 1 **Repetition in possible recombination events**. The data provided represent the repeated units in possible recombination events in Alydidae and Malcidae. Click here for file

Additional file 2 **Matrices of pairwise similarities within each type of *****tRNA***. The data provided represent the matrices of pairwise similarities within each type of *tRNA *used to identify the homologous gene. Click here for file

Additional file 3 **Different evolutionary patterns among genes**. The data provided represent different evolutionary patterns among genes, including nucleotide substitution number per site, CG content. Click here for file

Additional file 4 **Codon usage in each mt-genome**. The data provided represent the codon usage in each mt-genome. The most frequently used codons are indicated in bold. Click here for file

Additional file 5 **Tandem repetition analysis of control region**. The data provided represent the repeated units in control regions of mt-genomes. Click here for file

Additional file 6 **A+T content analysis of control region of Plataspidae**. The data provided represent the nucleotide composition of the control region of mt-genome of Plataspidae. Click here for file

Additional file 7 **Primers used in this study**. The data provided represent primers used in amplification of mt-genomes. Click here for file

## References

[B1] San Mauro D, Gower DJ, Zardoya R, Wilkinson M (2006). A hotspot of gene order rearrangement by tandem duplication and random loss in the vertebrate mitochondrial genome. Mol Biol Evol.

[B2] Dellaporta SL, Xu A, Sagasser S, Jakob W, Moreno MA, Buss LW, Schierwater B (2006). Mitochondrial genome of *Trichoplax adhaerens *supports Placozoa as the basal lower metazoan phylum. Proc Natl Acad Sci USA.

[B3] Boore JL (2001). Complete mitochondrial genome sequence of the polychaete annelid *Platynereis dumerilii*. Mol Biol Evol.

[B4] Boore JL, Brown WM (1998). Big trees from little genomes: mitochondrial gene order as a phylogenetic tool. Curr Opin Genet Dev.

[B5] Larget B, Simon DL, Kadane JB (2002). Bayesian phylogenetic inference from animal mitochondrial genome arrangements. Journal of the Royal Statistical Society Series B-Statistical Methodology.

[B6] Cameron SL, Beckenbach AT, Dowton M, Whiting MF (2006). Evidence from mitochondrial genomics on interordinal relationships in insects. Arthropod Systematics & Phylogeny.

[B7] Clary DO, Wolstenholme DR (1985). The mitochondrial DNA molecular of *Drosophila yakuba*: nucleotide sequence, gene organization, and genetic code. J Mol Evol.

[B8] Covacin C, Shao R, Cameron S, Barker SC (2006). Extraordinary number of gene rearrangements in the mitochondrial genomes of lice (Phthiraptera: Insecta). Insect Mol Biol.

[B9] Shao R, Barker SC (2003). The highly rearranged mitochondrial genome of the plague thrips, *Thrips imaginis *(Insecta: Thysanoptera): convergence of two novel gene boundaries and an extraordinary arrangement of rRNA genes. Mol Biol Evol.

[B10] Shao R, Campbell NJ, Barker SC (2001). Numerous gene rearrangements in the mitochondrial genome of the wallaby louse, *Heterodoxus macropus *(Phthiraptera). Mol Biol Evol.

[B11] Shao R, Campbell NJ, Schmidt ER, Barker SC (2001). Increased rate of gene rearrangement in the mitochondrial genomes of three orders of hemipteroid insects. Mol Biol Evol.

[B12] Thao ML, Baumann L, Baumann P (2004). Organization of the mitochondrial genomes of whiteflies, aphids, and psyllids (Hemiptera, Sternorrhyncha). BMC Evol Biol.

[B13] Štys P, Kerzhner I (1975). The rank and nomenclature of higher taxa in recent Heteroptera. Acta EntBohemoslov.

[B14] Schaefer CW (1993). The Pentatomomorpha (Hemiptera: Heteroptera): an annotated outline of its systematic history. EurJEntomol.

[B15] Schuh RT, Slater JA (1995). True Bugs of the World (Hemiptera: Heteroptera) Classification and Natural History.

[B16] Sweet MH, Schaefer CW (1996). The Comparative External Morphology of the Pregenital Abdomen of Hemiptera. Studies on Hemipteran Phylogeny.

[B17] Henry TJ (1997). Phylogenetic analysis of family groups within the infraorder Pentatomomorpha (Hemiptera: Heteroptera), with emphasis on the Lygaeoidea. AnnEntomolSocAm.

[B18] Xie Q, Bu W, Zheng L (2005). The Bayesian phylogenetic analysis of the 18S rRNA sequences from the main lineages of Trichophora (Insecta: Heteroptera: Pentatomomorpha). Mol Phylogenet Evol.

[B19] Li HM, Deng RQ, Wang JW, Chen ZY, Jia FL, Wang XZ (2005). A preliminary phylogeny of the Pentatomomorpha (Hemiptera: Heteroptera) based on nuclear 18S rDNA and mitochondrial DNA sequences. Mol Phylogenet Evol.

[B20] Wheeler WC, Schuh RT, Bang R (1993). Cladistic relationships among higher groups of Heteroptera: congruence between morphological and molecular data sets. EntomolScand.

[B21] Leston D (1958). Chromsome number and the systematics of Pentatomomorpha (Hemiptera). Proceedings of the Tenth International Congress of Entomologey.

[B22] Mattern MY (2004). Molecular phylogeny of the Gasterosteidae: the importance of using multiple genes. Mol Phylogenet Evol.

[B23] Miya M, Nishida M (2000). Use of mitogenomic information in teleostean molecular phylogenetics: a tree-based exploration under the maximum-parsimony optimality criterion. Mol Phylogenet Evol.

[B24] Wortley AH, Rudall PJ, Harris DJ, Scotland RW (2005). How much data are needed to resolve a difficult phylogeny? Case study in Lamiales. Syst Biol.

[B25] Shadel GS, Clayton DA (1997). Mitochondrial DNA maintenance in vertebrates. Annu Rev Biochem.

[B26] Tsaousis AD, Martin DP, Ladoukakis ED, Posada D, Zouros E (2005). Widespread recombination in published animal mtDNA sequences. Mol Biol Evol.

[B27] Kurabayashi A, Sumida M, Yonekawa H, Glaw F, Vences M, Hasegawa M (2008). Phylogeny, recombination, and mechanisms of stepwise mitochondrial genome reorganization in mantellid frogs from Madagascar. Mol Biol Evol.

[B28] Shao R, Dowton M, Murrell A, Barker SC (2003). Rates of gene rearrangement and nucleotide substitution are correlated in the mitochondrial genomes of insects. Mol Biol Evol.

[B29] Xu W, Jameson D, Tang B, Higgs PG (2006). The relationship between the rate of molecular evolution and the rate of genome rearrangement in animal mitochondrial genomes. Journal of Molecular Evolution.

[B30] Boore JL (1999). Animal mitochondrial genomes. Nucleic Acids Res.

[B31] Gibb GC, Kardailsky O, Kimball RT, Braun EL, Penny D (2007). Mitochondrial genomes and avian phylogeny: complex characters and resolvability without explosive radiations. Mol Biol Evol.

[B32] Dotson EM, Beard CB (2001). Sequence and organization of the mitochondrial genome of the Chagas disease vector, *Triatoma dimidiata*. Insect Mol Biol.

[B33] Boore JL, Lavrov DV, Brown WM (1998). Gene translocation links insects and crustaceans. Nature.

[B34] Mueller RL, Boore JL (2005). Molecular mechanisms of extensive mitochondrial gene rearrangement in plethodontid salamanders. Mol Biol Evol.

[B35] Dowton M, Austin AD (1999). Evolutionary dynamics of a mitochondrial rearrangement "hot spot" in the Hymenoptera. Mol Biol Evol.

[B36] Stanton DJ, Daehler LL, Moritz CC, Brown WM (1994). Sequences with the potential to form stem-and-loop structures are associated with coding-region duplications in animal mitochondrial DNA. Genetics.

[B37] Yu DJ, Xu L, Nardi F, Li JG, Zhang RJ (2007). The complete nucleotide sequence of the mitochondrial genome of the oriental fruit fly, Bactrocera dorsalis (Diptera: Tephritidae). Gene.

[B38] Lavrov DV, Lang BF (2005). Transfer RNA gene recruitment in mitochondrial DNA. Trends Genet.

[B39] Rawlings TA, Collins TM, Bieler R (2003). Changing identities: tRNA duplication and remolding within animal mitochondrial genomes. Proc Natl Acad Sci USA.

[B40] Štys P (1967). Monograph of Malcinae, with consideration of morphology and phylogeny of related groups (Heteroptera: Malcidae). Acta Entomol Mus Natl Prag.

[B41] Schuh RT, Štys P (1991). Phylogenetic analysis of cimicomorphan family relationships (Heteroptera). Journal Of The New York Entomological Society.

[B42] Zwickl DJ, Hillis DM (2002). Increased taxon sampling greatly reduces phylogenetic error. Systematic Biology.

[B43] Kim I, Cha SY, Yoon MH, Hwang JS, Lee SM, Sohn HD, Jin BR (2005). The complete nucleotide sequence and gene organization of the mitochondrial genome of the oriental mole cricket, *Gryllotalpa orientalis *(Orthoptera: Gryllotalpidae). Gene.

[B44] Graur D, Li WH (1999). Fundamentals of Molecular Evolution.

[B45] Saccone C, De Giorgi C, Gissi C, Pesole G, Reyes A (1999). Evolutionary genomics in Metazoa: the mitochondrial DNA as a model system. Gene.

[B46] Saccone C, Gissi C, Lanave C, Larizza A, Pesole G, Reyes A (2000). Evolution of the mitochondrial genetic system: an overview. Gene.

[B47] Roques S, Fox CJ, Villasana MI, Rico C (2006). The complete mitochondrial genome of the whiting, Merlangius merlangus and the haddock, Melanogrammus aeglefinus: a detailed genomic comparison among closely related species of the Gadidae family. Gene.

[B48] Broughton RE, Milam JE, Roe BA (2001). The complete sequence of the zebrafish (*Danio rerio*) mitochondrial genome and evolutionary patterns in vertebrate mitochondrial DNA. Genome Res.

[B49] Yukuhiro K, Sezutsu H, Itoh M, Shimizu K, Banno Y (2002). Significant levels of sequence divergence and gene rearrangements have occurred between the mitochondrial genomes of the wild mulberry silkmoth, *Bombyx mandarina*, and its close relative, the domesticated silkmoth, Bombyx mori. Mol Biol Evol.

[B50] Ballard JW (2000). Comparative genomics of mitochondrial DNA in members of the *Drosophila melanogaster *subgroup. J Mol Evol.

[B51] Clary DO, Wolstenholme DR (1983). Genes for cytochrome c oxidase subunit I, URF2, and three tRNAs in *Drosophila *mitochondrial DNA. Nucleic Acids Res.

[B52] de Bruijn MH (1983). *Drosophila melanogaster *mitochondrial DNA, a novel organization and genetic code. Nature.

[B53] Ojala D, Montoya J, Attardi G (1981). tRNA punctuation model of RNA processing in human mitochondria. Nature.

[B54] Boore JL (2006). The complete sequence of the mitochondrial genome of *Nautilus macromphalus *(Mollusca: Cephalopoda). BMC Genomics.

[B55] Perna NT, Kocher TD (1995). Patterns of nucleotide composition at fourfold degenerate sites of animal mitochondrial genomes. J Mol Evol.

[B56] Cameron SL, Whiting MF (2007). Mitochondrial genomic comparisons of the subterranean termites from the Genus *Reticulitermes *(Insecta: Isoptera: Rhinotermitidae). Genome.

[B57] Gibson A, Gowri-Shankar V, Higgs PG, Rattray M (2005). A comprehensive analysis of mammalian mitochondrial genome base composition and improved phylogenetic methods. Mol Biol Evol.

[B58] Xia X (1996). Maximizing transcription efficiency causes codon usage bias. Genetics.

[B59] Lynch M (1997). Mutation accumulation in nuclear, organelle, and prokaryotic transfer RNA genes. Mol Biol Evol.

[B60] Adams KL, Palmer JD (2003). Evolution of mitochondrial gene content: gene loss and transfer to the nucleus. Mol Phylogenet Evol.

[B61] Podsiadlowski L, Braband A (2006). The complete mitochondrial genome of the sea spider *Nymphon gracile *(Arthropoda: Pycnogonida). BMC Genomics.

[B62] Lavrov DV, Brown WM, Boore JL (2000). A novel type of RNA editing occurs in the mitochondrial tRNAs of the centipede *Lithobius forficatus*. Proc Natl Acad Sci USA.

[B63] Boore JL, Brown WM (2000). Mitochondrial genomes of Galathealinum, Helobdella, and Platynereis: sequence and gene arrangement comparisons indicate that Pogonophora is not a phylum and Annelida and Arthropoda are not sister taxa. Mol Biol Evol.

[B64] Wang X, Lavrov DV (2007). Mitochondrial genome of thehomoscleromorph *Oscarella carmela *(Porifera, Demospongiae) reveals unexpected complexity in the common ancestor of sponges and other animals. Mol Biol Evol.

[B65] Cook CE (2005). The complete mitochondrial genome of the stomatopod crustacean *Squilla mantis*. BMC Genomics.

[B66] Reineke A, Karlovsky P, Zebitz CP (1998). Preparation and purification of DNA from insects for AFLP analysis. Insect Mol Biol.

[B67] Simon C, Frati F, Beckenbach A, Cresp B, Liu H, Flook P (1994). Evolution, weighting, and phylogenetic utility of mitochondrial gene sequences and a compilation of conserved polymerase chain reaction primers. Ann Entomol Soc Am.

[B68] Hall TA (1999). BioEdit: a user-friendly biological sequence alignment editor and analysis program for Windows 95/98/NT. Acids Symp Ser.

[B69] Thompson JD, Gibson TJ, Plewniak F, Jeanmougin F, Higgins DG (1997). The CLUSTAL_X windows interface: flexible strategies for multiple sequence alignment aided by quality analysis tools. Nucleic Acids Res.

[B70] Lowe TM, Eddy SR (1997). tRNAscan-SE: a program for improved detection of transfer RNA genes in genomic sequence. Nucleic Acids Res.

[B71] Kumar S (1996). Phyltest: phylogeny hypothesis testing software.

[B72] Tamura K, Dudley J, Nei M, Kumar S (2007). MEGA4: molecular evolutionary genetics analysis (MEGA) software version 4.0. Mol Biol Evol.

[B73] Rozas J, Sanchez-DelBarrio JC, Messeguer X, Rozas R (2003). DnaSP, DNA polymorphism analyses by the coalescent and other methods. Bioinformatics.

[B74] Xia X (2000). Data analysis in molecular biology and evolution.

[B75] Xia X, Xie Z (2001). DAMBE: software package for data analysis in molecular biology and evolution. J Hered.

[B76] Posada D, Crandall KA (1998). Modeltest: testing the model of DNA substitution. Bioinformatics.

[B77] Swofford DL PAUP*: Phylogenetic Analysis Using Parsimony (*and Other Methods).Version 4.

[B78] Huelsenbeck JP, Ronquist F (2001). MRBAYES: Bayesian inference of phylogenetic trees. Bioinformatics.

