# Supplementary material for: Comparative and phylogenomic studies on the mitochondrial genomes of Pentatomomorpha (Insecta: Hemiptera: Heteroptera)
Source: BMC Genomics. 2008 Dec 17;9:610. doi: 10.1186/1471-2164-9-610 (PMC2651891; doi:10.1186/1471-2164-9-610)
Supplement: Additional file 1 — Repetition in possible recombination events. The data provided represent the repeated units in possible recombination events in Alydidae and Malcidae. [file 1471-2164-9-610-S1.doc]

**Repetition involved in possible recombination events**

The secondary structures were reconstructed with RNAstructure version 4.2 (Mathews et al. 1999).

**Alydidae**

- **Dup.A1, 15253 - 15278**

TATAGTTCTTTCCACCCCCTTAAATA

- **Dup.A2, 235 - 260**

TATTTAAGGGGGTGGAAAGAACTATA


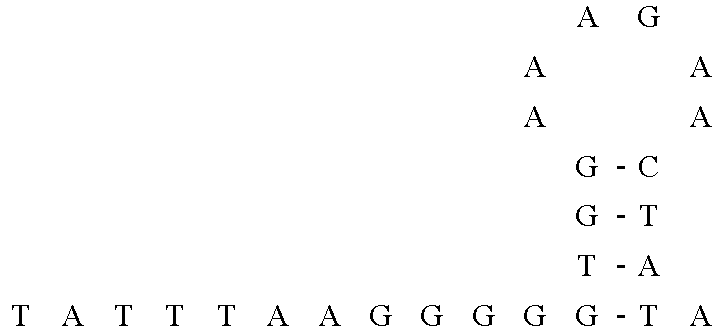


- **Dup.B1, 17131 - 17159**

TAAATAATTAATATAATATTATATAAATT

- **Dup.B2, 269 - 297**

TAAATAATTAATACAATATTATATAAATT

**Malcidae**

- **Dup.C1, 7891 – 7924; Dup.C2, 15538 - 15571**

ATATTTAACCCTTTTAAGAAAAAGAAAAAAAGGG


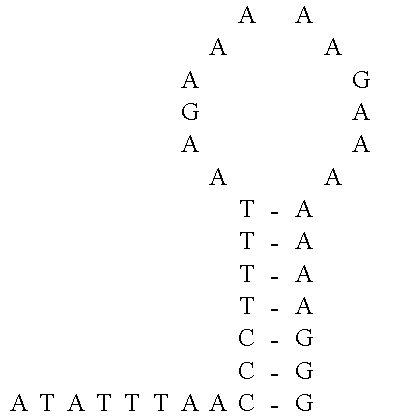


Mathews, D. H., J. Sabina, M. Zuker, and D. H. Turner. 1999. Expanded sequence dependence of thermodynamic parameters improves prediction of RNA secondary structure. J Mol Bio**l 2**88:911-940.
