# Supplementary material for: Comparative and phylogenomic studies on the mitochondrial genomes of Pentatomomorpha (Insecta: Hemiptera: Heteroptera)
Source: BMC Genomics. 2008 Dec 17;9:610. doi: 10.1186/1471-2164-9-610 (PMC2651891; doi:10.1186/1471-2164-9-610)
Supplement: Additional file 3 — Different evolutionary patterns among genes. The data provided represent different evolutionary patterns among genes, including nucleotide substitution number per site, CG content. [file 1471-2164-9-610-S3.doc]

**
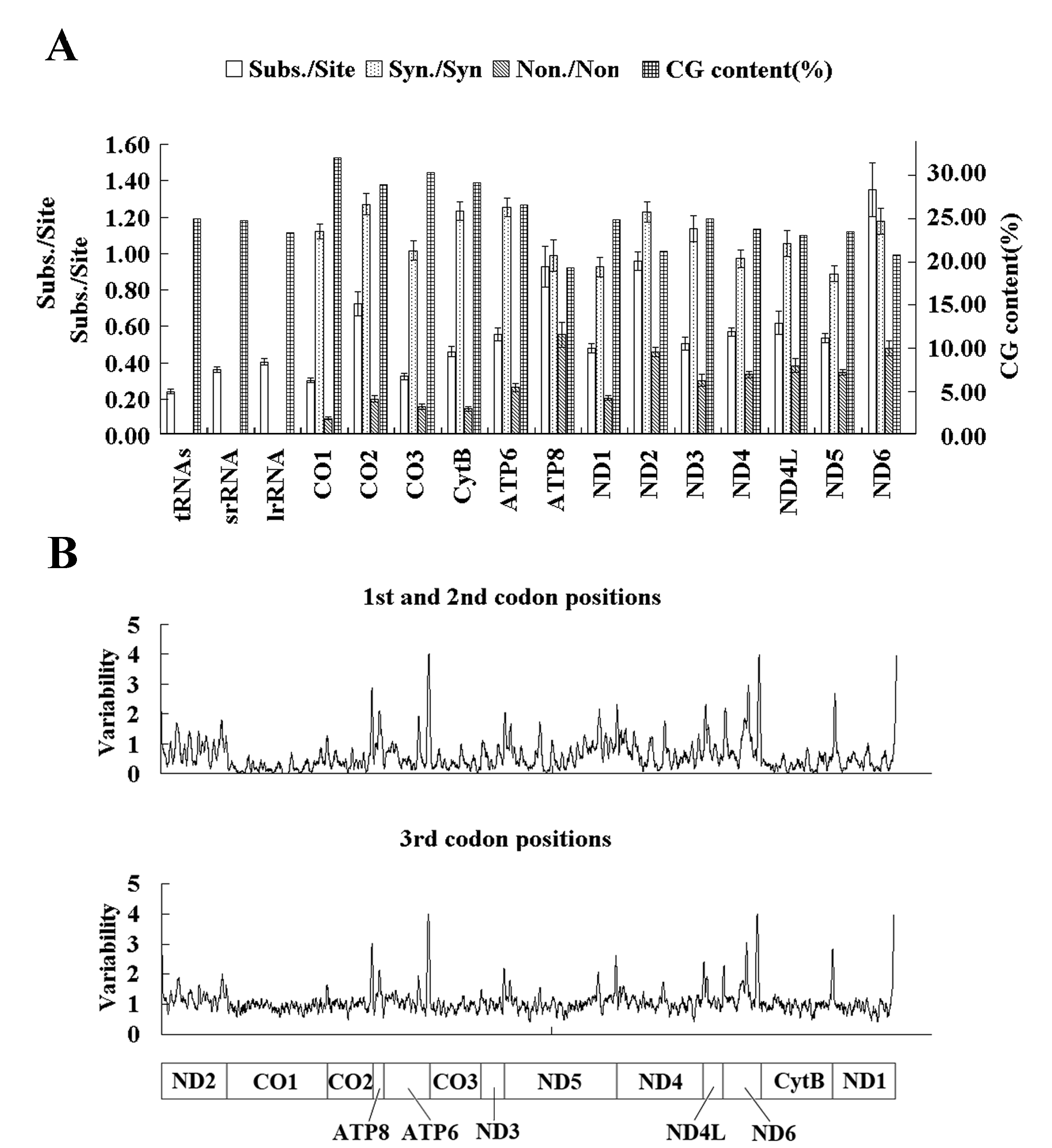
**

**Different evolutionary patterns among genes**. Panel A, the nucleotide substitution number per site from the averaging over all sequence pairs of each gene. The *tRNAs* were concatenated. The analyses were conducted using the Jukes-Cantor (Jukes and Cantor 1969) method. The standard error estimates were obtained by a bootstrap procedure (500 replicates) and the rate variation among sites was modeled with a gamma distribution estimated with the Modeltest. CG content and synonymous nucleotide substitutions per synonymous site and the nonsynonymous nucleotide substitutions per nonsynonymous site from the averaging over all sequence pairs were also presented. The analyses were conducted using the Kumar (Nei and Kumar 2000) method. The standard error estimates were obtained by a bootstrap procedure (500 replicates). Panel B, variability of the nucleotides across the concatenated protein coding genes. The overlapped nucleotides were treated independently. The curves were the mean values for all the 16 taxa with a sliding window of 20 nt for the first and the second codon positions and the 10 for the third codon positions.
