# Supplementary material for: Comparative and phylogenomic studies on the mitochondrial genomes of Pentatomomorpha (Insecta: Hemiptera: Heteroptera)
Source: BMC Genomics. 2008 Dec 17;9:610. doi: 10.1186/1471-2164-9-610 (PMC2651891; doi:10.1186/1471-2164-9-610)
Supplement: Additional file 5 — Tandem repetition analysis of control region. The data provided represent the repeated units in control regions of mt-genomes. [file 1471-2164-9-610-S5.pdf]

Alignment of repetitions (matches are noticed with vertical bar (|))

Alydidae

Positions 598....607(Query)

Period : 1 Exponent : 10.00 Size : 10 Error rate : 0.000 ACGT percentage: A : 100.00 C : 0.00 G : 0.00 T : 0.00

```
598 A
    |
599 A
    |
600 A
    |
601 A
    |
602 A
    |
603 A
    |
604 A
    |
605 A
    |
606 A
    |
607 A
```

Positions 641....651(Query)

Period : 1 Exponent : 11.00 Size : 11 Error rate : 0.000 ACGT percentage: A : 0.00 C : 0.00 G : 0.00 T : 100.00

```
641 T
    |
642 T
    |
643 T
    |
644 T
```

```
    |
645 T
    |
646 T
    |
647 T
    |
648 T
    |
649 T
    |
650 T
    |
651 T
```

Positions 654....678(Query)

Period : 10 Exponent : 2.50 Size : 25 Error rate : 0.000 ACGT percentage: A : 68.00 C : 16.00 G : 8.00 T : 8.00

```
654 AAAAAATCGC
    ||||||||||
664 AAAAAATCGC
    |||||
674 AAAAA
```

Positions 674....701(Query)

Period : 10 Exponent : 2.80 Size : 28 Error rate : 0.000 ACGT percentage: A : 60.71 C : 0.00 G : 0.00 T : 39.29

```
674 AAAAATTTTA
    ||||||||||
684 AAAAATTTTA
    ||||||||
694 AAAAATTT
```

Positions 715....740(Query)

Period : 11 Exponent : 2.36 Size : 26 Error rate : 0.000 ACGT percentage: A : 76.92 C : 7.69 G : 7.69 T : 7.69

```
715 AAAAAATCGAA
    |||||||||||
726 AAAAAATCGAA
    ||||
737 AAAA
```

Positions 744....781(Query)

Period : 10 Exponent : 3.80 Size : 38 Error rate : 0.000 ACGT percentage: A : 73.68 C : 7.89 G : 10.53 T : 7.89

```
744 GAAAAAAATC
    ||||||||||
754 GAAAAAAATC
    ||||||||||
764 GAAAAAAATC
    ||||||||
774 GAAAAAAA
```

Positions 823....844(Query)

Period : 10 Exponent : 2.20 Size : 22 Error rate : 0.000 ACGT percentage: A : 63.64 C : 0.00 G : 9.09 T : 27.27

```
823 TTGAAAAAAA
    ||||||||||
833 TTGAAAAAAA
    ||
843 TT
```

Positions 949....959(Query)

Period : 1 Exponent : 11.00 Size : 11 Error rate : 0.000 ACGT percentage: A : 0.00 C : 0.00 G : 0.00 T : 100.00

949 T
|
950 T
|
951 T
|
952 T
|
953 T
|
954 T
|
955 T
|
956 T
|
957 T
|
958 T
|
959 T

Positions 1069....1078(Query)

Period : 1 Exponent : 10.00 Size : 10 Error rate : 0.000 ACGT percentage: A : 100.00 C : 0.00 G : 0.00 T : 0.00

1069 A
|
1070 A
|
1071 A
|
1072 A
|
1073 A
|
1074 A

```
     |
1075 A
     |
1076 A
     |
1077 A
     |
1078 A
```

Positions 1093....1117(Query)

Period : 10 Exponent : 2.50 Size : 25 Error rate : 0.000 ACGT percentage: A : 68.00 C : 16.00 G : 8.00 T : 8.00

```
1093 AAAAATCGCA
     ||||||||||
1103 AAAAATCGCA
     |||||
1113 AAAAA
```

Positions 1146....1171(Query)

Period : 9 Exponent : 2.89 Size : 26 Error rate : 0.000 ACGT percentage: A : 57.69 C : 19.23 G : 11.54 T : 11.54

```
1146 AAAAATCGC
     |||||||||
1155 AAAAATCGC
     ||||||||
1164 AAAAATCG
```

Positions 1173....1198(Query)

Period : 10 Exponent : 2.60 Size : 26 Error rate : 0.000 ACGT percentage: A : 76.92 C : 7.69 G : 7.69 T : 7.69

```
1173 AAAAAATCGA
     ||||||||||
1183 AAAAAATCGA
     ||||||
1193 AAAAAA
```

Positions 1924....1936(Query)

Period : 3 Exponent : 4.33 Size : 13 Error rate : 0.000 ACGT percentage: A : 61.54 C : 0.00 G : 0.00 T : 38.46

```
1924 TAA
     |||
1927 TAA
     |||
1930 TAA
     |||
1933 TAA
     |
1936 T
```

Aradidae

Positions 335....350(Query)

Period : 5 Exponent : 3.20 Size : 16 Error rate : 0.000 ACGT percentage: A : 18.75 C : 0.00 G : 37.50 T : 43.75

```
335 TTGGA
    |||||
340 TTGGA
    |||||
345 TTGGA
    |
350 T
```

Positions 352....363(Query)

Period : 1 Exponent : 12.00 Size : 12 Error rate : 0.000 ACGT percentage: A : 0.00 C : 0.00 G : 100.00 T : 0.00

352 G
|
353 G
|
354 G
|
355 G
|
356 G
|
357 G
|
358 G
|
359 G
|
360 G
|
361 G
|
362 G
|
363 G

Berytidae

Positions 495....504(Query)

Period : 1 Exponent : 10.00 Size : 10 Error rate : 0.000 ACGT percentage: A : 0.00 C : 0.00 G : 0.00 T : 100.00

495 T
|
496 T
|
497 T
|
498 T

```
    |
499 T
    |
500 T
    |
501 T
    |
502 T
    |
503 T
    |
504 T
```

Positions 593....613(Query)

Period : 10 Exponent : 2.10 Size : 21 Error rate : 0.000 ACGT percentage: A : 42.86 C : 9.52 G : 0.00 T : 47.62

```
593 ATATTAATTC
    ||||||||||
603 ATATTAATTC
    |
613 A
```

Positions 696....1181(Query)

Period : 56 Exponent : 8.68 Size : 486 Error rate : 0.000 ACGT percentage: A : 43.00 C : 5.35 G : 3.50 T : 48.15

```
696  ATATTTAAATTTGTATAAATATAAATTTCTAAATATTCAATATTATTATTTCATAG
     ||||||||||||||||||||||||||||||||||||||||||||||||||||||||
752  ATATTTAAATTTGTATAAATATAAATTTCTAAATATTCAATATTATTATTTCATAG
     ||||||||||||||||||||||||||||||||||||||||||||||||||||||||
808  ATATTTAAATTTGTATAAATATAAATTTCTAAATATTCAATATTATTATTTCATAG
     ||||||||||||||||||||||||||||||||||||||||||||||||||||||||
864  ATATTTAAATTTGTATAAATATAAATTTCTAAATATTCAATATTATTATTTCATAG
     ||||||||||||||||||||||||||||||||||||||||||||||||||||||||
920  ATATTTAAATTTGTATAAATATAAATTTCTAAATATTCAATATTATTATTTCATAG
     ||||||||||||||||||||||||||||||||||||||||||||||||||||||||
```

```
976   ATATTTAAATTTGTATAAATATAAATTTCTAAATATTCAATATTATTATTTCATAG
      ||||||||||||||||||||||||||||||||||||||||||||||||||||||||
1032 ATATTTAAATTTGTATAAATATAAATTTCTAAATATTCAATATTATTATTTCATAG
      ||||||||||||||||||||||||||||||||||||||||||||||||||||||||
1088 ATATTTAAATTTGTATAAATATAAATTTCTAAATATTCAATATTATTATTTCATAG
      ||||||||||||||||||||||||||||||||||||||
1144 ATATTTAAATTTGTATAAATATAAATTTCTAAATATTC
```

Coreidae

Positions 764....777(Query)

Period : 5 Exponent : 2.80 Size : 14 Error rate : 0.000 ACGT percentage: A : 64.29 C : 14.29 G : 0.00 T : 21.43

```
764 AAATC
    |||||
769 AAATC
    ||||
774 AAAT
```

Positions 1104....1113(Query)

Period : 1 Exponent : 10.00 Size : 10 Error rate : 0.000 ACGT percentage: A : 100.00 C : 0.00 G : 0.00 T : 0.00

```
1104 A
     |
1105 A
     |
1106 A
     |
1107 A
     |
1108 A
     |
1109 A
     |
1110 A
```

```
     |
1111 A
     |
1112 A
     |
1113 A
```

Largidae

Positions 1....146(Query)

Period : 62 Exponent : 2.35 Size : 146 Error rate : 0.000 ACGT percentage: A : 38.36 C : 18.49 G : 10.27 T : 32.88

```
1   ATATTAATAGCAGGAACCTCCTTCTCCTCTATGTTATTTATAGGAATAAAACTACAATATAA
    ||||||||||||||||||||||||||||||||||||||||||||||||||||||||||||||
63  ATATTAATAGCAGGAACCTCCTTCTCCTCTATGTTATTTATAGGAATAAAACTACAATATAA
    ||||||||||||||||||||||
125 ATATTAATAGCAGGAACCTCCT
```

Malcidae

Positions 650....662(Query)

Period : 4 Exponent : 3.25 Size : 13 Error rate : 0.000 ACGT percentage: A : 46.15 C : 0.00 G : 0.00 T : 53.85

```
650 TTAA
    ||||
654 TTAA
    ||||
658 TTAA
    |
662 T
```

Positions 879....893(Query)

Period : 4 Exponent : 3.75 Size : 15 Error rate : 0.000 ACGT percentage: A : 53.33 C : 0.00 G : 0.00 T : 46.67

```
879 AATT
    ||||
883 AATT
    ||||
887 AATT
    |||
891 AAT
```

Pentatomidae

Wed Mar 26 08:23:37 2008

Positions 740....972(Query)

Period : 63 Exponent : 3.70 Size : 233 Error rate : 0.000 ACGT percentage: A : 48.07 C : 8.58 G : 6.44 T : 36.91

```
740 ATAATTATTAGTTAATTAATAATCACAGATAATAAGATTTAATAACCATATATACTGATATCA
    |||||||||||||||||||||||||||||||||||||||||||||||||||||||||||||||
803 ATAATTATTAGTTAATTAATAATCACAGATAATAAGATTTAATAACCATATATACTGATATCA
    |||||||||||||||||||||||||||||||||||||||||||||||||||||||||||||||
866 ATAATTATTAGTTAATTAATAATCACAGATAATAAGATTTAATAACCATATATACTGATATCA
    ||||||||||||||||||||||||||||||||||||||||||||
929 ATAATTATTAGTTAATTAATAATCACAGATAATAAGATTTAATA
```

Positions 978....1165(Query)

Period : 63 Exponent : 2.98 Size : 188 Error rate : 0.000 ACGT percentage: A : 47.87 C : 10.64 G : 6.38 T : 35.11

```
978  ATATACTGATATCAATAATTATTAGTTAATTAATAATCACAGATAATAAGATTTAATAACCAC
     |||||||||||||||||||||||||||||||||||||||||||||||||||||||||||||||
1041 ATATACTGATATCAATAATTATTAGTTAATTAATAATCACAGATAATAAGATTTAATAACCAC
     ||||||||||||||||||||||||||||||||||||||||||||||||||||||||||||||
```

```
1104  ATATACTGATATCAATAATTATTAGTTAATTAATAATCACAGATAATAAGATTTAATAACCA
```

Positions 1104....1354(Query)

Period : 63 Exponent : 3.98 Size : 251 Error rate : 0.000 ACGT percentage: A : 47.81 C : 9.56 G : 6.37 T : 36.25

```
1104  ATATACTGATATCAATAATTATTAGTTAATTAATAATCACAGATAATAAGATTTAATAACCAT
      |||||||||||||||||||||||||||||||||||||||||||||||||||||||||||||||
1167  ATATACTGATATCAATAATTATTAGTTAATTAATAATCACAGATAATAAGATTTAATAACCAT
      |||||||||||||||||||||||||||||||||||||||||||||||||||||||||||||||
1230  ATATACTGATATCAATAATTATTAGTTAATTAATAATCACAGATAATAAGATTTAATAACCAT
      ||||||||||||||||||||||||||||||||||||||||||||||||||||||||||||||
1293  ATATACTGATATCAATAATTATTAGTTAATTAATAATCACAGATAATAAGATTTAATAACCA
```

Positions 1293....1431(Query)

Period : 63 Exponent : 2.21 Size : 139 Error rate : 0.000 ACGT percentage: A : 46.76 C : 11.51 G : 6.47 T : 35.25

```
1293  ATATACTGATATCAATAATTATTAGTTAATTAATAATCACAGATAATAAGATTTAATAACCAC
      |||||||||||||||||||||||||||||||||||||||||||||||||||||||||||||||
1356  ATATACTGATATCAATAATTATTAGTTAATTAATAATCACAGATAATAAGATTTAATAACCAC
      ||||||||||||
1419  ATATACTGATATC
```

Positions 1653....1666(Query)

Period : 4 Exponent : 3.50 Size : 14 Error rate : 0.000 ACGT percentage: A : 50.00 C : 0.00 G : 0.00 T : 50.00

```
1653  ATTA
      ||||
1657  ATTA
      ||||
1661  ATTA
```

||
1665 AT

Positions 1716....1729(Query)

Period : 4 Exponent : 3.50 Size : 14 Error rate : 0.000 ACGT percentage: A : 50.00 C : 0.00 G : 0.00 T : 50.00

1716 ATTA
||||
1720 ATTA
||||
1724 ATTA
||
1728 AT

Positions 1836....2134(Query)

Period : 126 Exponent : 2.37 Size : 299 Error rate : 0.000 ACGT percentage: A : 46.49 C : 10.03 G : 7.69 T : 35.79

1836
ATAATTATTAGTTAATTAATAATCACAGATAATAAGATTTAATAACCATATATACTGATATCGATAATTATTAGT
TAATTAATAATCACAGATAATAAGATTTAATAACCACATATACTGATATCG

||||||||||||||||||||||||||||||||||||||||||||||||||||||||||||||||||||||||||||
|||||||||||||||||||||||||||||||||||||||||||||||||||
1962
ATAATTATTAGTTAATTAATAATCACAGATAATAAGATTTAATAACCATATATACTGATATCGATAATTATTAGT
TAATTAATAATCACAGATAATAAGATTTAATAACCACATATACTGATATCG
|||||||||||||||||||||||||||||||||||||||||||||||
2088 ATAATTATTAGTTAATTAATAATCACAGATAATAAGATTTAATAACC

Positions 2166....2190(Query)

Period : 12 Exponent : 2.08 Size : 25 Error rate : 0.000 ACGT percentage: A : 52.00 C : 16.00 G : 0.00 T : 32.00

```
2166 ATTAATAATCAC
     ||||||||||||
2178 ATTAATAATCAC
     |
2190 A
```

Plataspidae

Positions 56....83(Query)

Period : 13 Exponent : 2.15 Size : 28 Error rate : 0.000 ACGT percentage: A : 0.00 C : 60.71 G : 0.00 T : 39.29

```
56 TCCTCCTCTCCTC
   |||||||||||||
69 TCCTCCTCTCCTC
   ||
82 TC
```

Positions 58....73(Query)

Period : 5 Exponent : 3.20 Size : 16 Error rate : 0.000 ACGT percentage: A : 0.00 C : 62.50 G : 0.00 T : 37.50

```
58 CTCCT
   |||||
63 CTCCT
   |||||
68 CTCCT
   |
73 C
```

Positions 63....81(Query)

Period : 8 Exponent : 2.38 Size : 19 Error rate : 0.000 ACGT percentage: A : 0.00 C : 63.16 G : 0.00 T : 36.84

```
63 CTCCTCTC
   ||||||||
71 CTCCTCTC
   |||
79 CTC
```

Positions 343....356(Query)

Period : 2 Exponent : 7.00 Size : 14 Error rate : 0.000 ACGT percentage: A : 50.00 C : 0.00 G : 0.00 T : 50.00

```
343 AT
    ||
345 AT
    ||
347 AT
    ||
349 AT
    ||
351 AT
    ||
353 AT
    ||
355 AT
```

Positions 373....384(Query)

Period : 1 Exponent : 12.00 Size : 12 Error rate : 0.000 ACGT percentage: A : 0.00 C : 0.00 G : 100.00 T : 0.00

```
373 G
    |
374 G
    |
375 G
    |
376 G
    |
```

377 G
|
378 G
|
379 G
|
380 G
|
381 G
|
382 G
|
383 G
|
384 G

Positions 499....1000(Query)

Period : 117 Exponent : 4.29 Size : 502 Error rate : 0.000 ACGT percentage: A : 44.82 C : 9.36 G : 7.17 T : 38.65

499
AATTTAAAAAGTAAAAATATTGCTACTGGACTAAATTATATATTAGCATATATATCTCTCTCACAGAATACAATA
AATTTAAATAAATTAGCAGATTTTAATTATTAATTAATTTTT

|||||||||||||||||||||||||||||||||||||||||||||||||||||||||||||||||||||||||||
||||||||||||||||||||||||||||||||||||||||||
616
AATTTAAAAAGTAAAAATATTGCTACTGGACTAAATTATATATTAGCATATATATCTCTCTCACAGAATACAATA
AATTTAAATAAATTAGCAGATTTTAATTATTAATTAATTTTT

|||||||||||||||||||||||||||||||||||||||||||||||||||||||||||||||||||||||||||
||||||||||||||||||||||||||||||||||||||||||
733
AATTTAAAAAGTAAAAATATTGCTACTGGACTAAATTATATATTAGCATATATATCTCTCTCACAGAATACAATA
AATTTAAATAAATTAGCAGATTTTAATTATTAATTAATTTTT

|||||||||||||||||||||||||||||||||||||||||||||||||||||||||||||||||||||||||||
||||||||||||||||||||||||||||||||||||||||||
850
AATTTAAAAAGTAAAAATATTGCTACTGGACTAAATTATATATTAGCATATATATCTCTCTCACAGAATACAATA
AATTTAAATAAATTAGCAGATTTTAATTATTAATTAATTTTT

||||||||||||||||||||||||||||||||||
967 AATTTAAAAAGTAAAAATATTGCTACTGGACTAA

Positions 1012....1025(Query)

Period : 2 Exponent : 7.00 Size : 14 Error rate : 0.000 ACGT percentage: A : 50.00 C : 0.00 G : 0.00 T : 50.00

1012 AT
||
1014 AT
||
1016 AT
||
1018 AT
||
1020 AT
||
1022 AT
||
1024 AT

Pyrrhocoridae

Positions 194....457(Query)

Period : 87 Exponent : 3.03 Size : 264 Error rate : 0.000 ACGT percentage: A : 29.55 C : 28.03 G : 5.68 T : 36.74

194
CCTTATAGATTAACTAACACTCTCTGTTCATTACCGAACTACTACTCTTTCCTCCAACCAAATAGTTGCATATAT
TATACTTTATCC

|||||||||||||||||||||||||||||||||||||||||||||||||||||||||||||||||||||||||||
||||||||||||
281
CCTTATAGATTAACTAACACTCTCTGTTCATTACCGAACTACTACTCTTTCCTCCAACCAAATAGTTGCATATAT
TATACTTTATCC

```
|||||||||||||||||||||||||||||||||||||||||||||||||||||||||||||||||||||||||||
||||||||||||
368
CCTTATAGATTAACTAACACTCTCTGTTCATTACCGAACTACTACTCTTTCCTCCAACCAAATAGTTGCATATAT
TATACTTTATCC
    |||
455 CCT
```

Positions 504....524(Query)

Period : 10 Exponent : 2.10 Size : 21 Error rate : 0.000 ACGT percentage: A : 38.10 C : 9.52 G : 0.00 T : 52.38

```
504 TCTAAAATTT
    ||||||||||
514 TCTAAAATTT
    |
524 T
```

Positions 637....647(Query)

Period : 2 Exponent : 5.50 Size : 11 Error rate : 0.000 ACGT percentage: A : 45.45 C : 0.00 G : 0.00 T : 54.55

```
637 TA
    ||
639 TA
    ||
641 TA
    ||
643 TA
    ||
645 TA
    |
647 T
```

Positions 651....663(Query)

Period : 4   Exponent : 3.25   Size : 13   Error rate : 0.000   ACGT percentage: A : 76.92   C : 0.00   G : 0.00   T : 23.08

```
651 AAAT
    ||||
655 AAAT
    ||||
659 AAAT
    |
663 A
```

Positions 683....808(Query)

Period : 36   Exponent : 3.50   Size : 126   Error rate : 0.000   ACGT percentage: A : 63.49   C : 11.11   G : 3.17   T : 22.22

```
683 ATAAAATCATGTACAAAACAATATAAAAATCATAAA
    ||||||||||||||||||||||||||||||||||||
719 ATAAAATCATGTACAAAACAATATAAAAATCATAAA
    ||||||||||||||||||||||||||||||||||||
755 ATAAAATCATGTACAAAACAATATAAAAATCATAAA
    ||||||||||||||||||
791 ATAAAATCATGTACAAAA
```

Positions 846....941(Query)

Period : 36   Exponent : 2.67   Size : 96   Error rate : 0.000   ACGT percentage: A : 65.62   C : 7.29   G : 2.08   T : 25.00

```
846 AATATAAAAATCATAAAATAAAATCATGTACAAAAT
    ||||||||||||||||||||||||||||||||||||
882 AATATAAAAATCATAAAATAAAATCATGTACAAAAT
    ||||||||||||||||||||||||
918 AATATAAAAATCATAAAATAAAAT
```

Positions 923....1304(Query)

Period : 188 Exponent : 2.03 Size : 382 Error rate : 0.000 ACGT percentage: A : 66.75 C : 6.81 G : 1.57 T : 24.87

923
AAAAATCATAAAATAAAATTATTACAAAATAATAAAAAAATAAAATAAAATCATAAAATAAAATCATGTACAAAA
TAATATAAAAATCATAAAATAAAATCATGTACAAAATAATATAAAAATCATAAAATAAAATCATGTACAAAATAA
TATAAAAATCATAAAATAAAATTATTACAAAATAATAA

1111
AAAAATCATAAAATAAAATTATTACAAAATAATAAAAAAATAAAATAAAATCATAAAATAAAATCATGTACAAAA
TAATATAAAAATCATAAAATAAAATCATGTACAAAATAATATAAAAATCATAAAATAAAATCATGTACAAAATAA
TATAAAAATCATAAAATAAAATTATTACAAAATAATAA
1299 AAAAAT

Positions 959....973(Query)

Period : 5 Exponent : 3.00 Size : 15 Error rate : 0.000 ACGT percentage: A : 80.00 C : 0.00 G : 0.00 T : 20.00

959 AAAAT
|||||
964 AAAAT
|||||
969 AAAAT

Positions 962....989(Query)

Period : 13 Exponent : 2.15 Size : 28 Error rate : 0.000 ACGT percentage: A : 67.86 C : 7.14 G : 0.00 T : 25.00

962 ATAAAATAAAATC

```
     |||||||||||||
975  ATAAAATAAAATC
     ||
988  AT
```

Positions 969....1094(Query)

Period : 36 Exponent : 3.50 Size : 126 Error rate : 0.000 ACGT percentage: A : 65.08 C : 7.94 G : 2.38 T : 24.60

```
969   AAAATCATAAAATAAAATCATGTACAAAATAATATA
      ||||||||||||||||||||||||||||||||||||
1005  AAAATCATAAAATAAAATCATGTACAAAATAATATA
      ||||||||||||||||||||||||||||||||||||
1041  AAAATCATAAAATAAAATCATGTACAAAATAATATA
      ||||||||||||||||||
1077  AAAATCATAAAATAAAAT
```

Positions 1076....1151(Query)

Period : 35 Exponent : 2.17 Size : 76 Error rate : 0.000 ACGT percentage: A : 69.74 C : 5.26 G : 0.00 T : 25.00

```
1076  AAAAATCATAAAATAAAATTATTACAAAATAATAA
      |||||||||||||||||||||||||||||||||||
1111  AAAAATCATAAAATAAAATTATTACAAAATAATAA
      ||||||
1146  AAAAAT
```

Positions 1147....1161(Query)

Period : 5 Exponent : 3.00 Size : 15 Error rate : 0.000 ACGT percentage: A : 80.00 C : 0.00 G : 0.00 T : 20.00

```
1147  AAAAT
      |||||
```

```
1152 AAAAT
     |||||
1157 AAAAT
```

Positions 1150....1177(Query)

Period : 13 Exponent : 2.15 Size : 28 Error rate : 0.000 ACGT percentage: A : 67.86 C : 7.14 G : 0.00 T : 25.00

```
1150 ATAAAATAAAATC
     |||||||||||||
1163 ATAAAATAAAATC
     ||
1176 AT
```

Positions 1157....1282(Query)

Period : 36 Exponent : 3.50 Size : 126 Error rate : 0.000 ACGT percentage: A : 65.08 C : 7.94 G : 2.38 T : 24.60

```
1157 AAAATCATAAAATAAAATCATGTACAAAATAATATA
     ||||||||||||||||||||||||||||||||||||
1193 AAAATCATAAAATAAAATCATGTACAAAATAATATA
     ||||||||||||||||||||||||||||||||||||
1229 AAAATCATAAAATAAAATCATGTACAAAATAATATA
     ||||||||||||||||||
1265 AAAATCATAAAATAAAAT
```

Positions 1300....1314(Query)

Period : 5 Exponent : 3.00 Size : 15 Error rate : 0.000 ACGT percentage: A : 80.00 C : 0.00 G : 0.00 T : 20.00

```
1300 AAAAT
     |||||
1305 AAAAT
```

```
     |||||
1310 AAAAT
```

Reduviidae

Positions 437....449(Query)

Period : 1 Exponent : 13.00 Size : 13 Error rate : 0.000 ACGT percentage: A : 0.00 C : 0.00 G : 100.00 T : 0.00

```
437 G
    |
438 G
    |
439 G
    |
440 G
    |
441 G
    |
442 G
    |
443 G
    |
444 G
    |
445 G
    |
446 G
    |
447 G
    |
448 G
    |
449 G
```

Positions 1118....1549(Query)

Period : 140 Exponent : 3.09 Size : 432 Error rate : 0.000 ACGT percentage: A : 40.51 C : 22.92 G : 15.51 T : 21.06

1118
GGACATCCGGAATAAACAAAATATAACCGGACAATCATAATCTATGAATCTAGACTAAATAAAATCTTAAACTCA
CCCCTTTCTGATAAAGAGGACCAAAATATCGTACCACCCTGTACAATCGCAGGAATACATGCGGG

|||||||||||||||||||||||||||||||||||||||||||||||||||||||||||||||||||||||||||
|||||||||||||||||||||||||||||||||||||||||||||||||||||||||||||||||
1258
GGACATCCGGAATAAACAAAATATAACCGGACAATCATAATCTATGAATCTAGACTAAATAAAATCTTAAACTCA
CCCCTTTCTGATAAAGAGGACCAAAATATCGTACCACCCTGTACAATCGCAGGAATACATGCGGG

|||||||||||||||||||||||||||||||||||||||||||||||||||||||||||||||||||||||||||
|||||||||||||||||||||||||||||||||||||||||||||||||||||||||||||||||
1398
GGACATCCGGAATAAACAAAATATAACCGGACAATCATAATCTATGAATCTAGACTAAATAAAATCTTAAACTCA
CCCCTTTCTGATAAAGAGGACCAAAATATCGTACCACCCTGTACAATCGCAGGAATACATGCGGG
     ||||||||||||
1538 GGACATCCGGAA

Saldidae

Positions 378....389(Query)

Period : 2 Exponent : 6.00 Size : 12 Error rate : 0.000 ACGT percentage: A : 50.00 C : 0.00 G : 0.00 T : 50.00

378 TA
    ||
380 TA
    ||
382 TA
    ||
384 TA
    ||
386 TA
    ||
388 TA
