# Supplementary material for: Comparative and phylogenomic studies on the mitochondrial genomes of Pentatomomorpha (Insecta: Hemiptera: Heteroptera)
Source: BMC Genomics. 2008 Dec 17;9:610. doi: 10.1186/1471-2164-9-610 (PMC2651891; doi:10.1186/1471-2164-9-610)

Percentage of A+T content along the J-strand of the putative control region of Plataspidae. With the sliding window of 50 nucleotides. The shadowed part was the tandemly repeated (117 nt)$_4$.

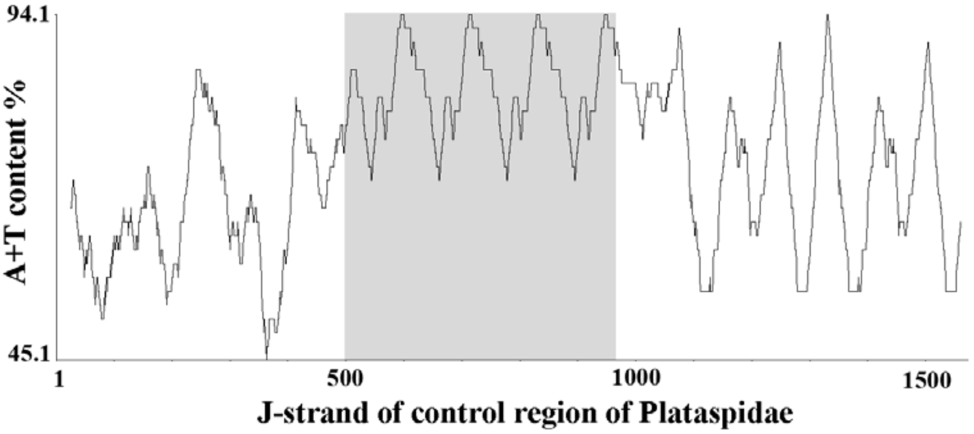

Supplement: Additional file 6 — A+T content analysis of control region of Plataspidae. The data provided represent the nucleotide composition of the control region of mt-genome of Plataspidae. [file 1471-2164-9-610-S6.pdf]
